# Supplementary figures and images for: Netrin signaling mediates survival of dormant epithelial ovarian cancer cells
Source: eLife. 2024 Jul 18;12:RP91766. doi: 10.7554/eLife.91766 (PMC11257678; doi:10.7554/eLife.91766)

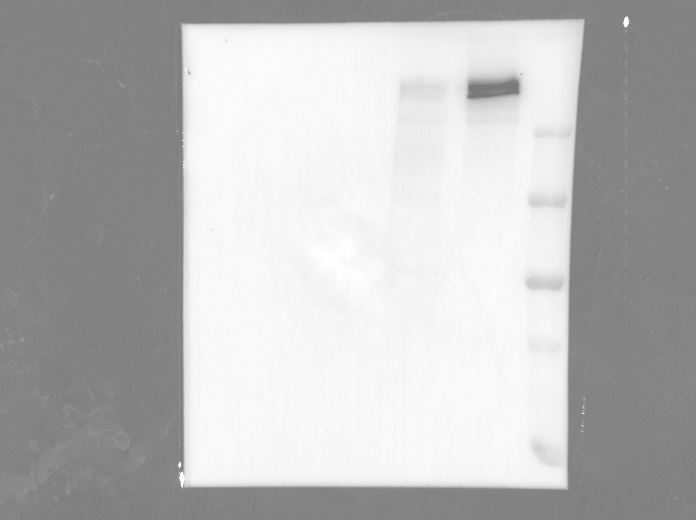

Supplement: Figure 1—source data 1. [file elife-91766-fig1-data1.zip › Ki67 OVCAR8 2023-11-21 14hr 13min_Exposure_4.0sec+user 2023-11-21 14hr 10min - Copy.tif]

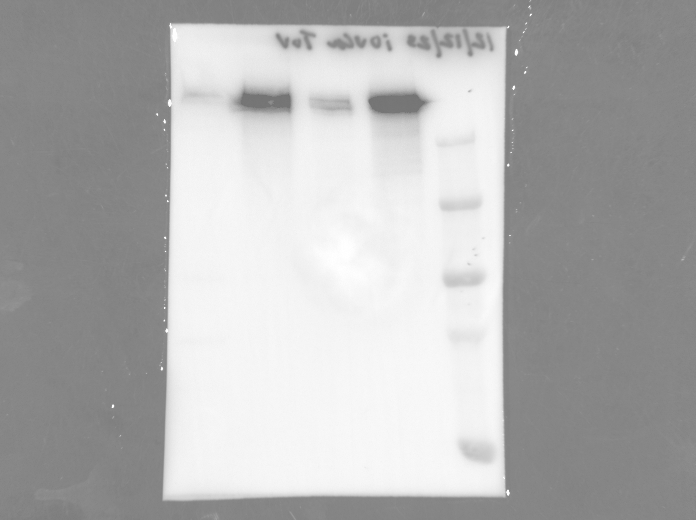

Supplement: Figure 1—source data 1. [file elife-91766-fig1-data1.zip › Ki67 TOV_iOvCa147 2023-12-13 14hr 03min_Exposure_9.0sec+user 2023-12-13 13hr 59min - Copy.tif]

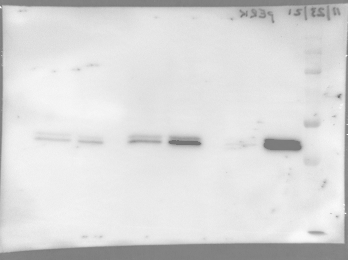

Supplement: Figure 1—source data 1. [file elife-91766-fig1-data1.zip › OVCAR8_TOV_OVCAR3 pERK 2021-11-24 13hr 22min_Exposure_6.0sec+user 2021-11-24 13hr 15min.tif]

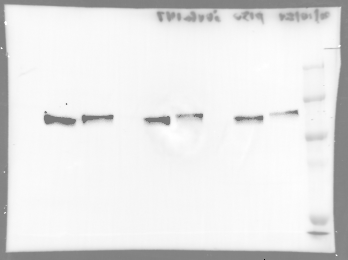

Supplement: Figure 1—source data 1. [file elife-91766-fig1-data1.zip › p130 iOvCa147 n2024-01-12 14hr 03min_Exposure_42.0sec+template for p130.tif]

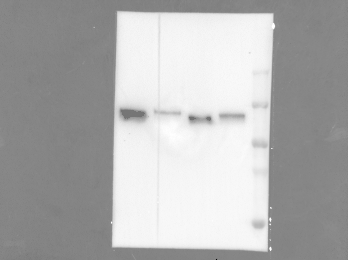

Supplement: Figure 1—source data 1. [file elife-91766-fig1-data1.zip › p130 iOvCa147_TOV1946 2023-12-13 13hr 55min_Exposure_42.0sec+template for p130.tif]

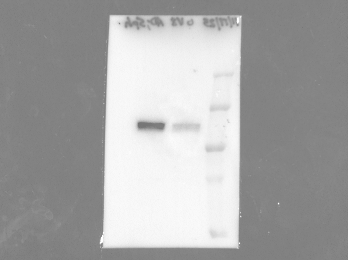

Supplement: Figure 1—source data 1. [file elife-91766-fig1-data1.zip › p130 OVCAR8 2023-11-21 14hr 19min_Exposure_8.0sec+user 2023-11-21 14hr 17min.tif]

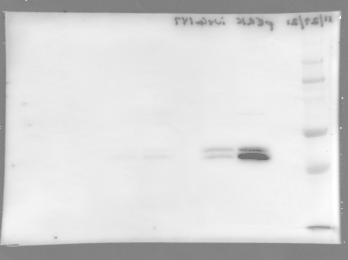

Supplement: Figure 1—source data 1. [file elife-91766-fig1-data1.zip › pERK iOvCa147_OVCAR32021-11-30 14hr 44min_Exposure_4.0sec+user 2021-11-30 14hr 43min.tif]

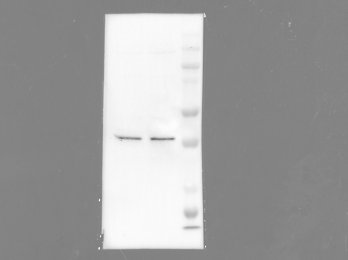

Supplement: Figure 1—source data 1. [file elife-91766-fig1-data1.zip › Phospho p38 2023-12-20 14hr 13min_Exposure_20.0sec+Template for Phospho p38.tif]

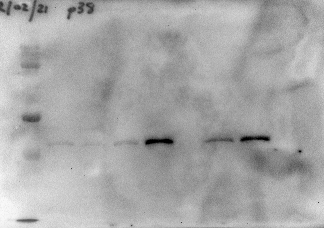

Supplement: Figure 1—source data 1. [file elife-91766-fig1-data1.zip › pP38 OVCAR8_TOV_iOvCa147_2.tif]

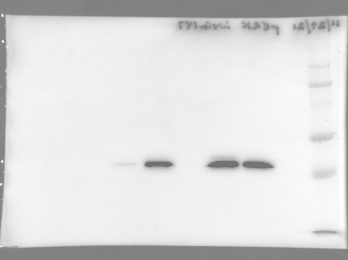

Supplement: Figure 1—source data 1. [file elife-91766-fig1-data1.zip › Total ERK iOvCa147 2021-12-01 14hr 52min-1_Exposure_6.0sec+user 2021-12-01 14hr 52min.tif]

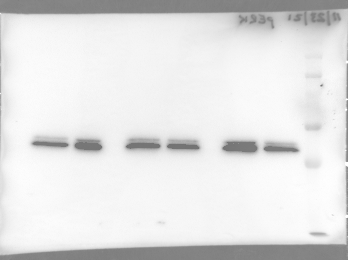

Supplement: Figure 1—source data 1. [file elife-91766-fig1-data1.zip › Total ERK OVCAR8_TOV19462021-11-25 12hr 42min_Exposure_8.0sec+user 2021-11-25 12hr 27min.tif]

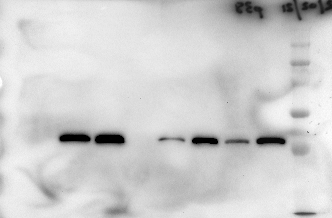

Supplement: Figure 1—source data 1. [file elife-91766-fig1-data1.zip › Total P38 2021-12-07 14hr 06min_Exposure_50.0sec+user 2021-12-07 14hr 03min.tif]

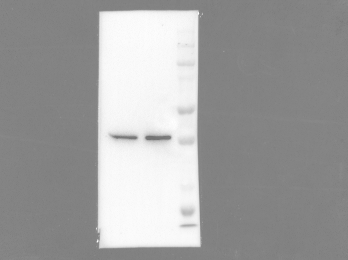

Supplement: Figure 1—source data 1. [file elife-91766-fig1-data1.zip › Total p38 2023-12-21 15hr 29min_Exposure_3.0sec+template for Total p38.tif]

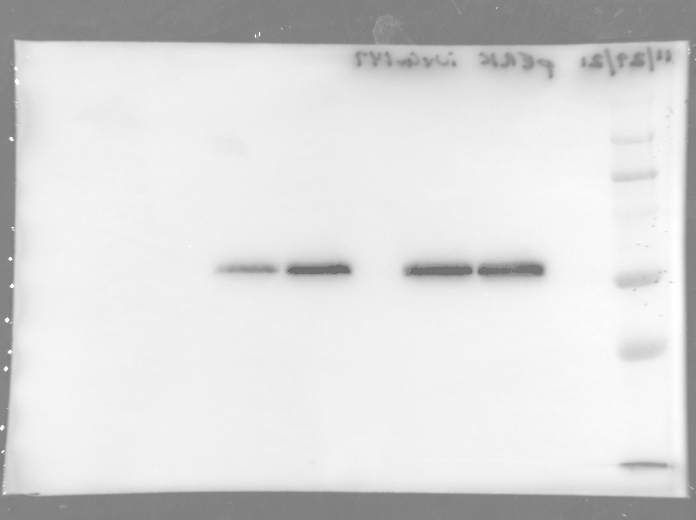

Supplement: Figure 1—source data 1. [file elife-91766-fig1-data1.zip › tubulin 2021-12-02 14hr iOvCa147.tif]

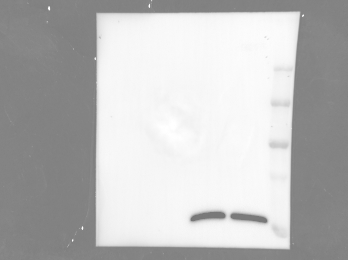

Supplement: Figure 1—source data 1. [file elife-91766-fig1-data1.zip › Tubulin for Ki67 OVCAR8 2023-12-19 15hr 44min_Exposure_4.0sec+user 2023-12-19 15hr 43min.tif]

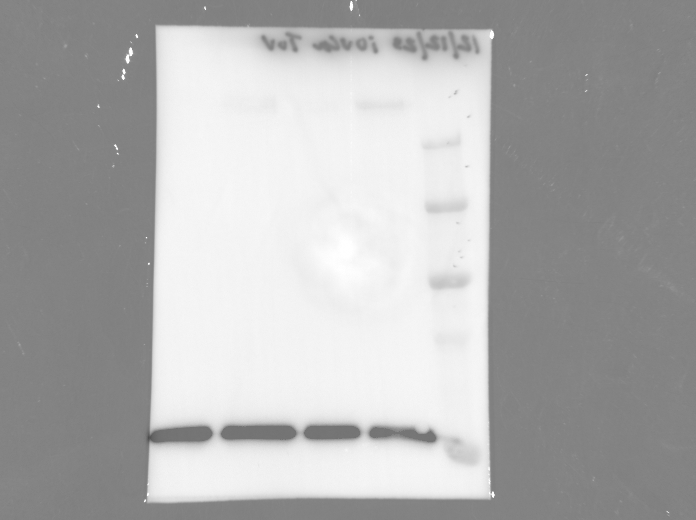

Supplement: Figure 1—source data 1. [file elife-91766-fig1-data1.zip › Tubulin for Ki67 TOV 2023-12-15 16hr 45min_Exposure_5.0sec+template for tubulin for p130.tif]

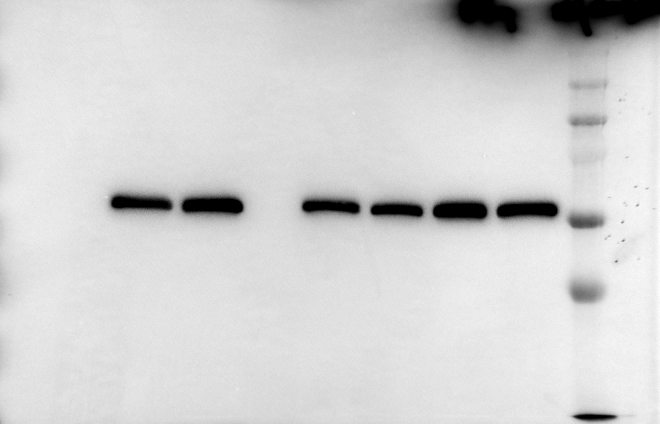

Supplement: Figure 1—source data 1. [file elife-91766-fig1-data1.zip › Tubulin FOR p38 2021-12-08 14hr 09min_Exposure_10.0sec+user 2021-12-08 14hr 08min.tif]

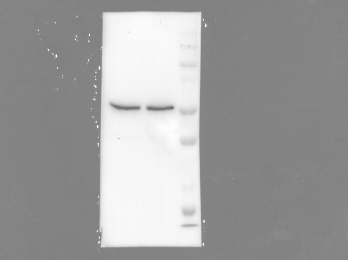

Supplement: Figure 1—source data 1. [file elife-91766-fig1-data1.zip › Tubulin for p38 2023-12-22 13hr 08min_Exposure_4.0sec+user 2023-12-22 13hr 07min.tif]

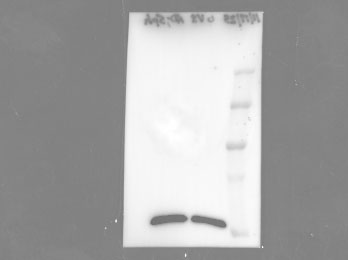

Supplement: Figure 1—source data 1. [file elife-91766-fig1-data1.zip › Tubulin for p130 2023-12-19 15hr 48min-1_Exposure_4.0sec+user 2023-12-19 15hr 48min.tif]

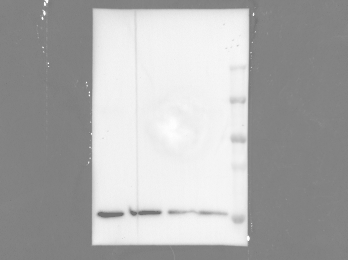

Supplement: Figure 1—source data 1. [file elife-91766-fig1-data1.zip › Tubulin for p130 TOV 2023-12-15 16hr 31min_Exposure_180.0sec+Template for tubulin Ki67.tif]

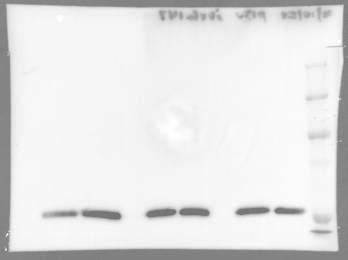

Supplement: Figure 1—source data 1. [file elife-91766-fig1-data1.zip › Tubulin iOvCa147 for p130 2024-01-16 15hr 32min_Exposure_20.0sec+user 2024-01-16 15hr 31min.tif]

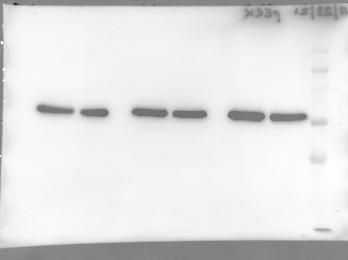

Supplement: Figure 1—source data 1. [file elife-91766-fig1-data1.zip › Tubulin OVCAR8_TOV1946 2021-11-26 14hr 47min_Exposure_4.0sec+user 2021-11-26 14hr 46min.tif]

Fig.1A

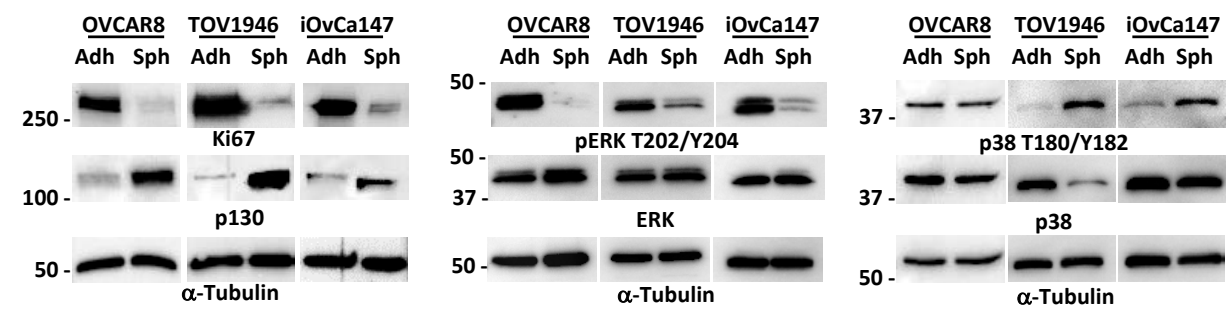

Fig.1A

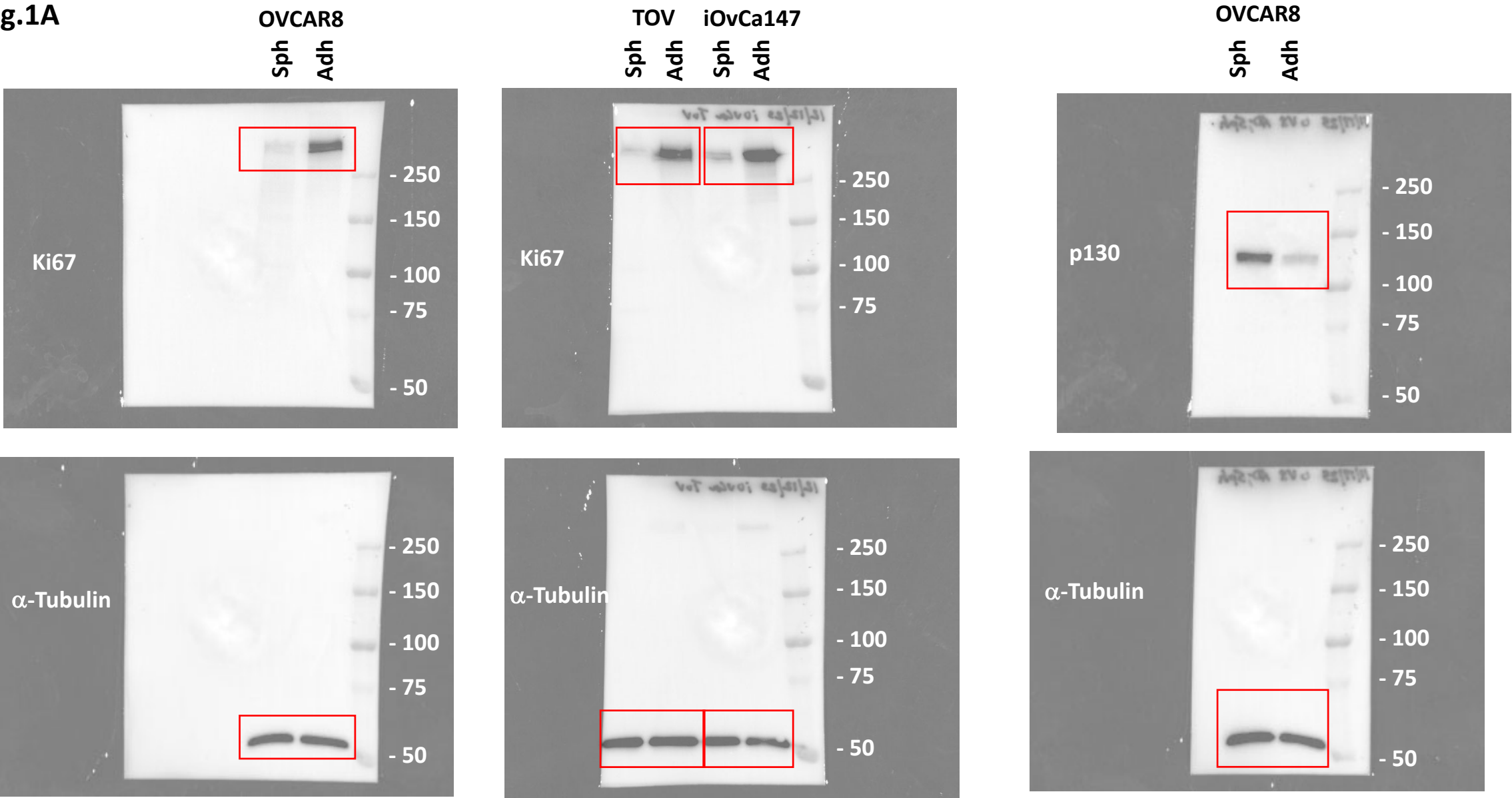

Fig.1A

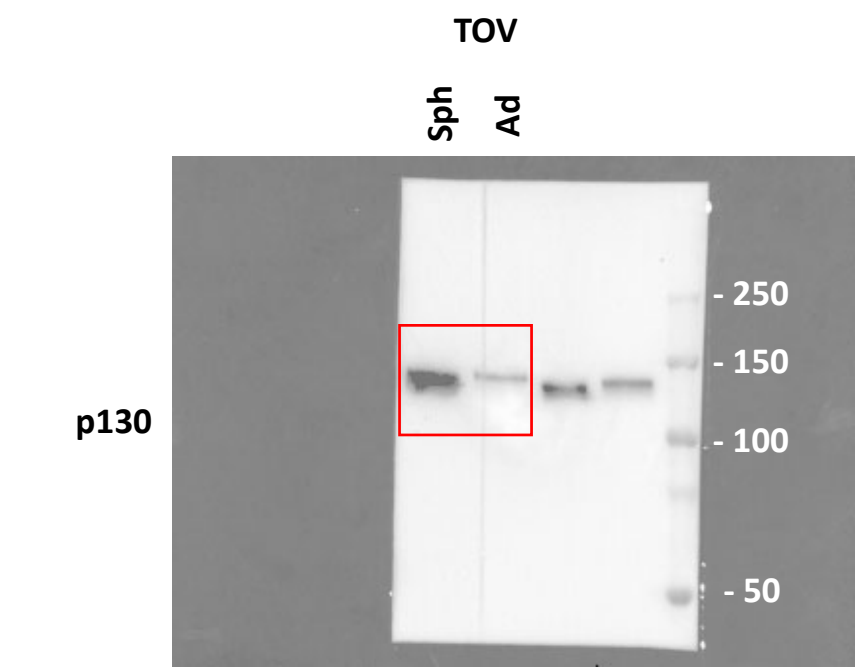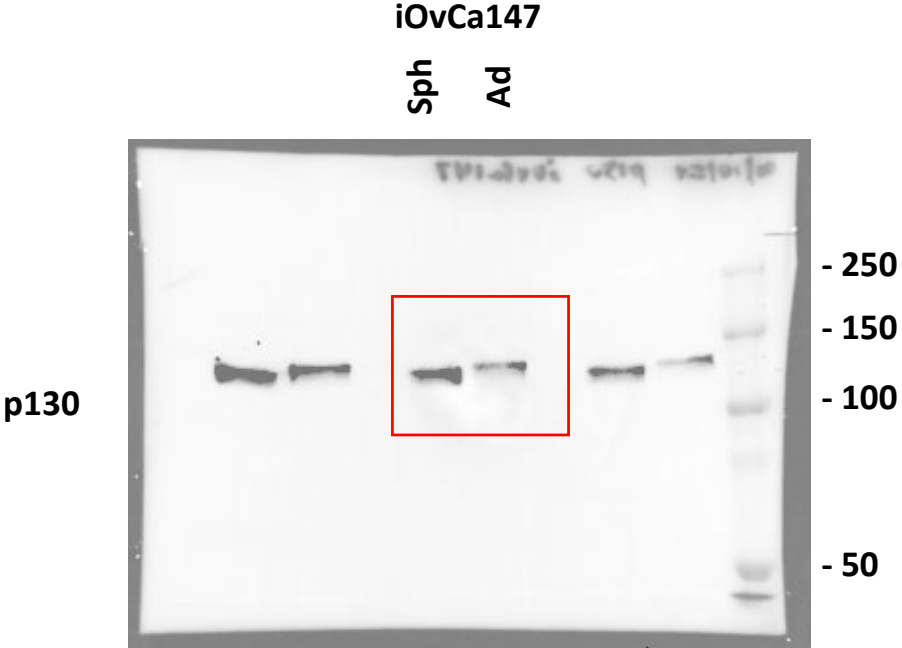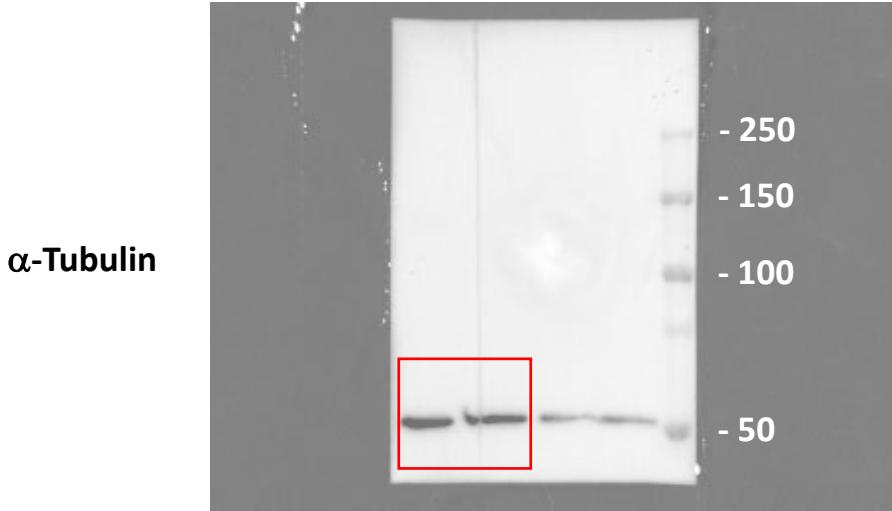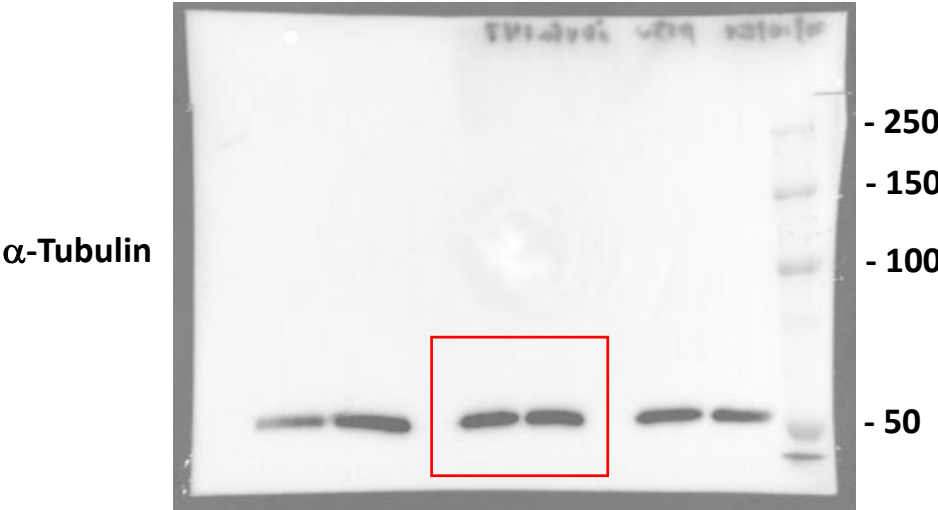

Fig.1A

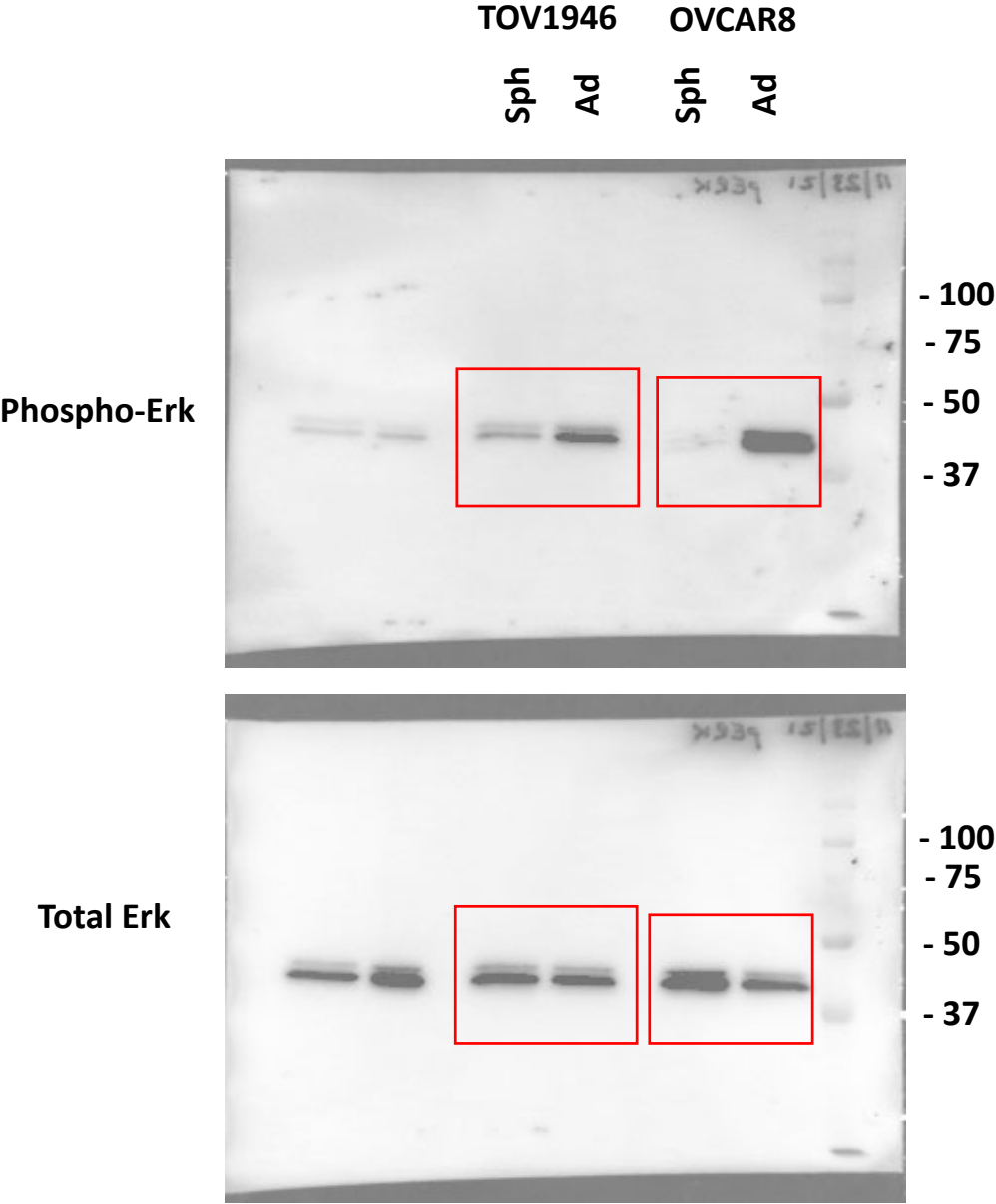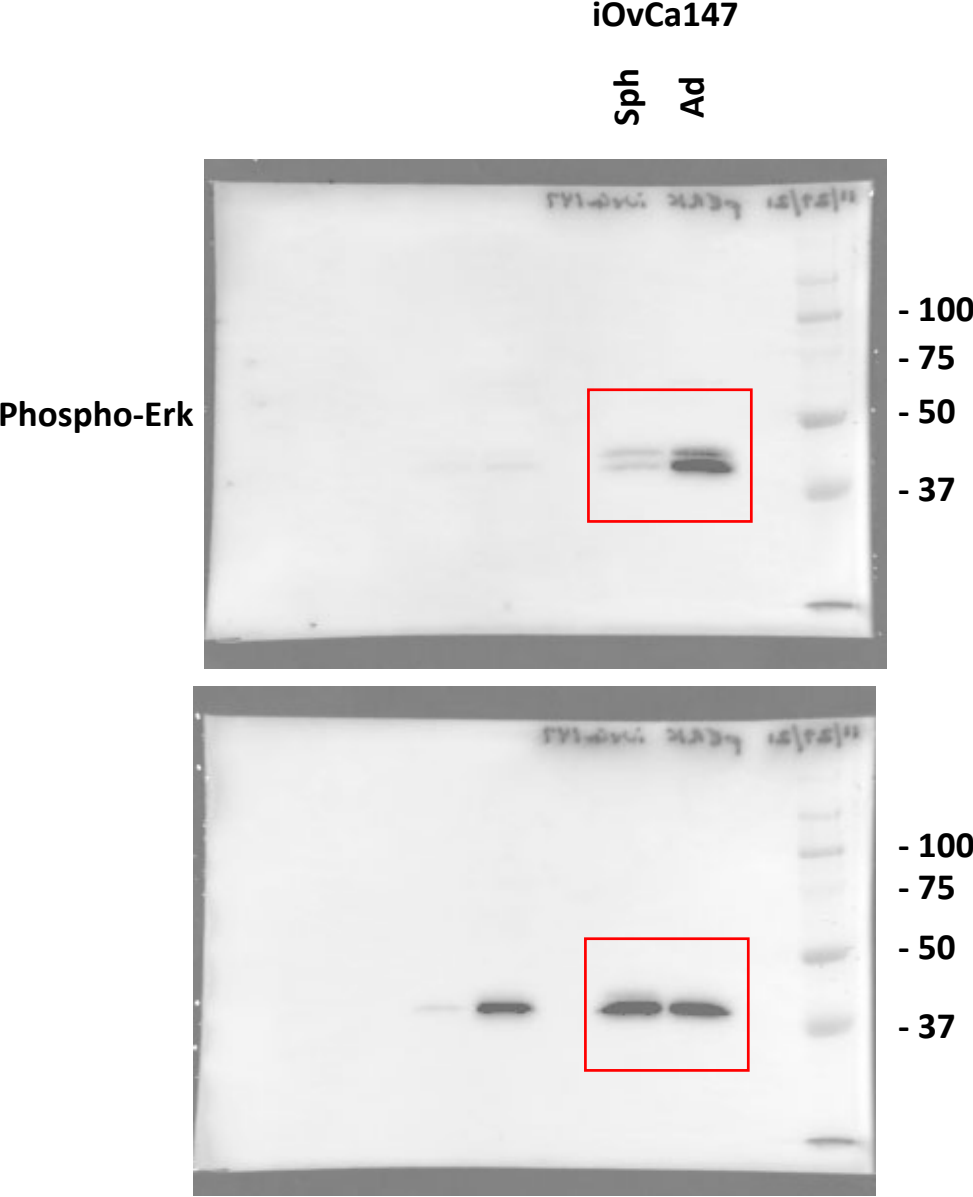

Fig.1A

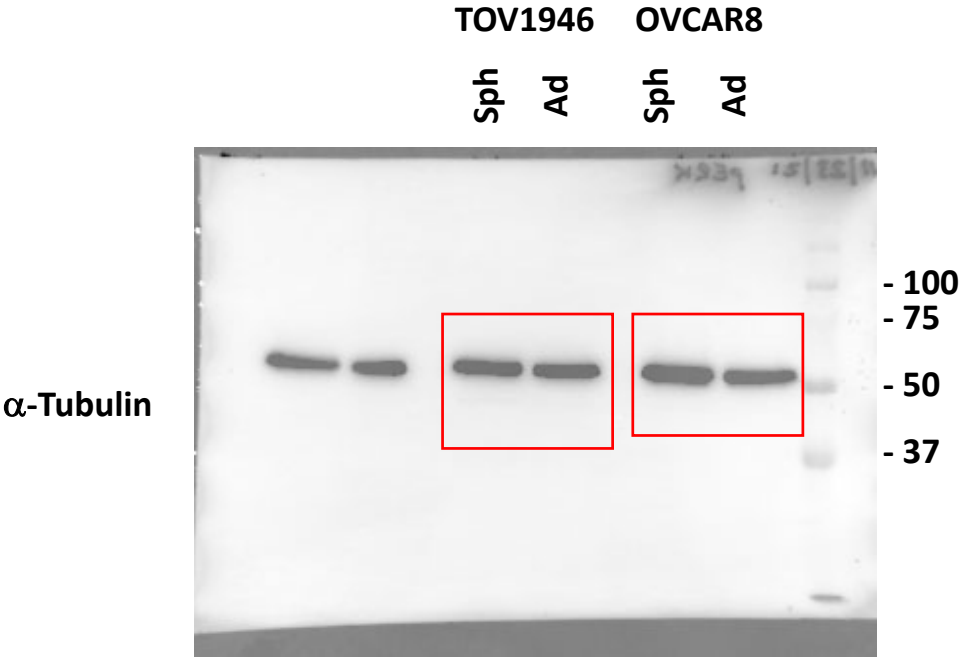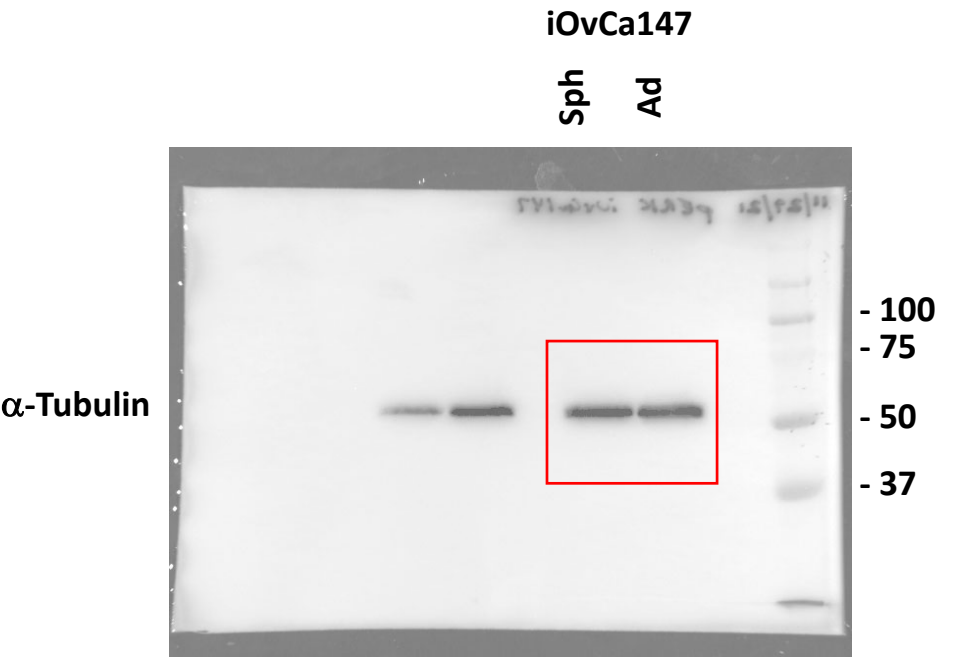

**Fig.1A**

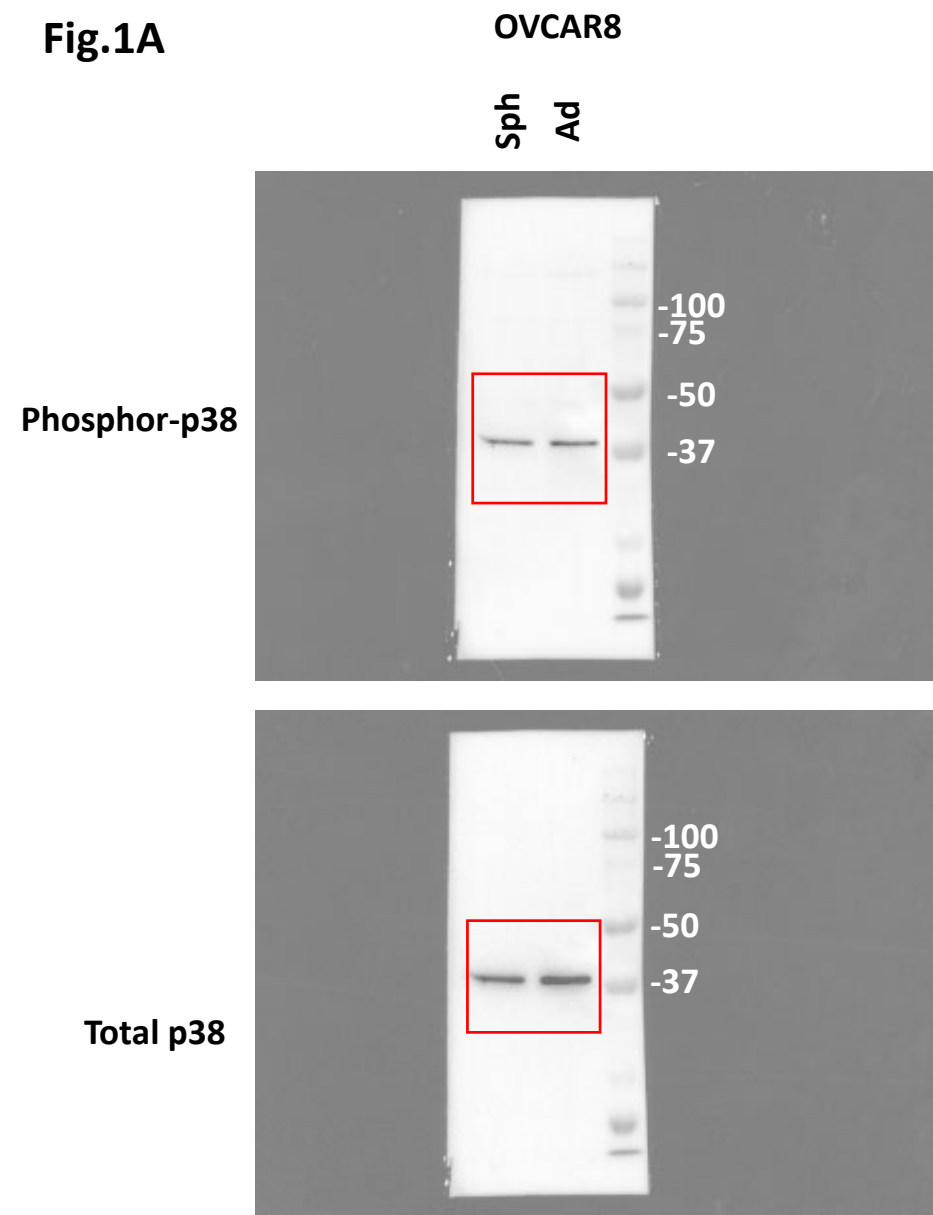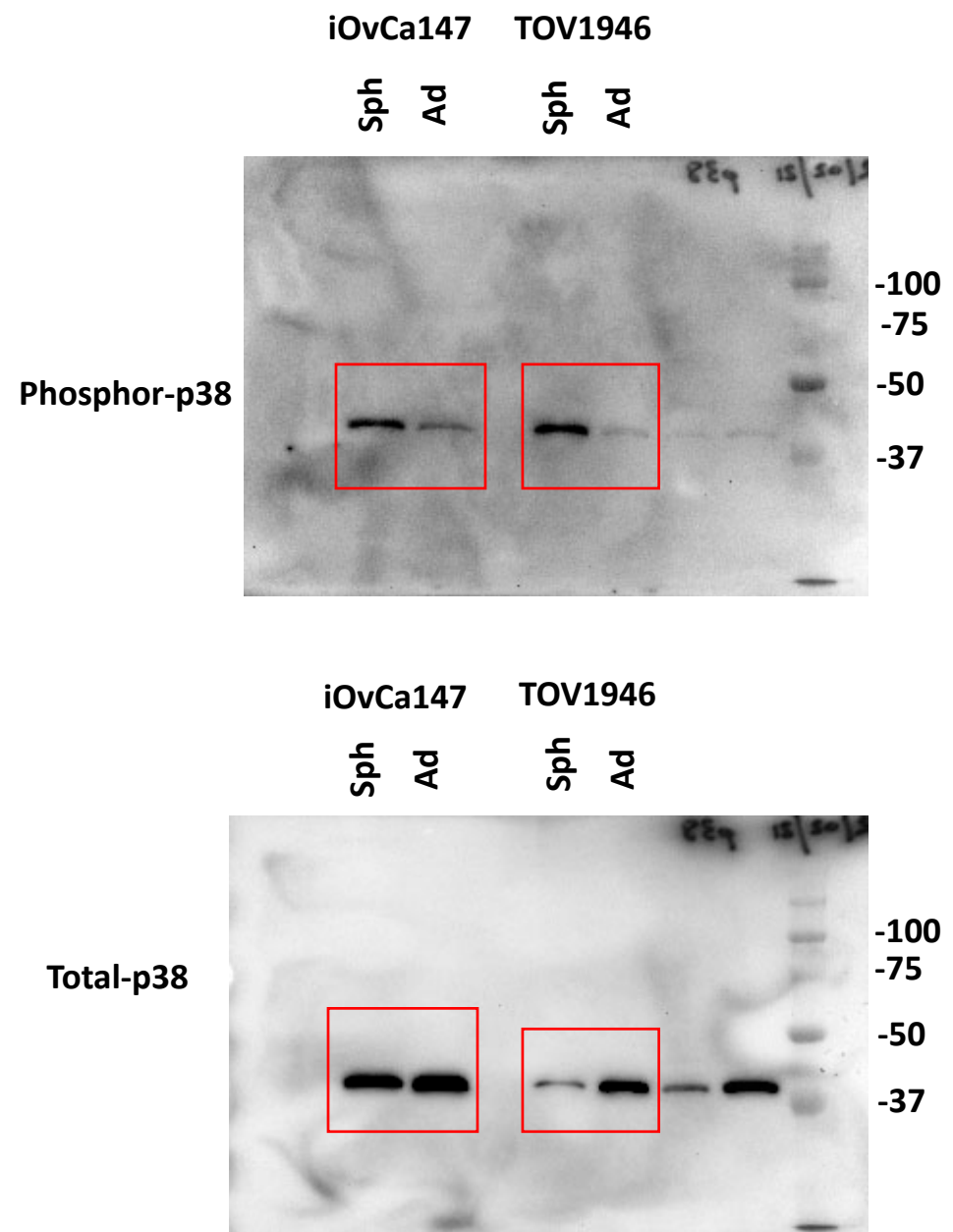

**Fig.1A**

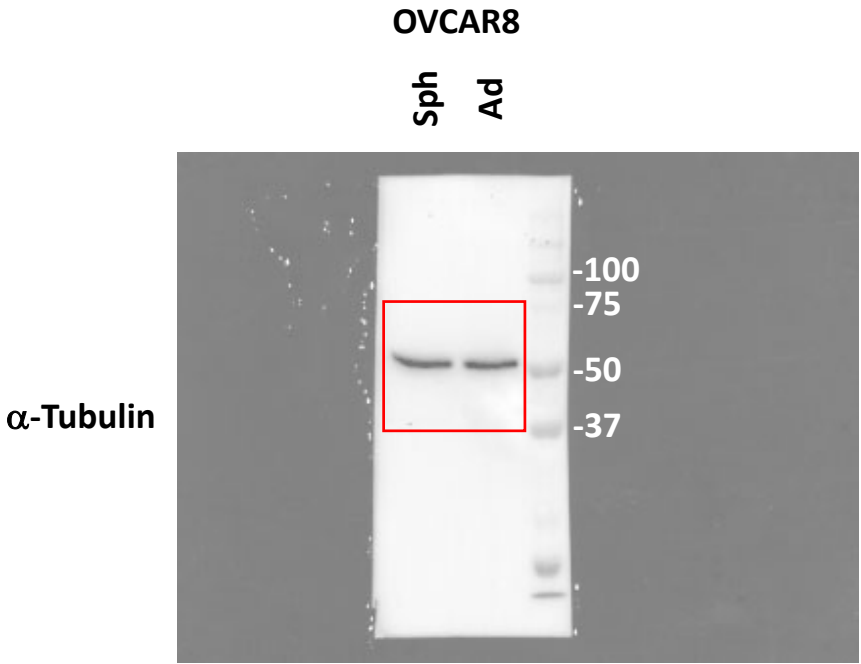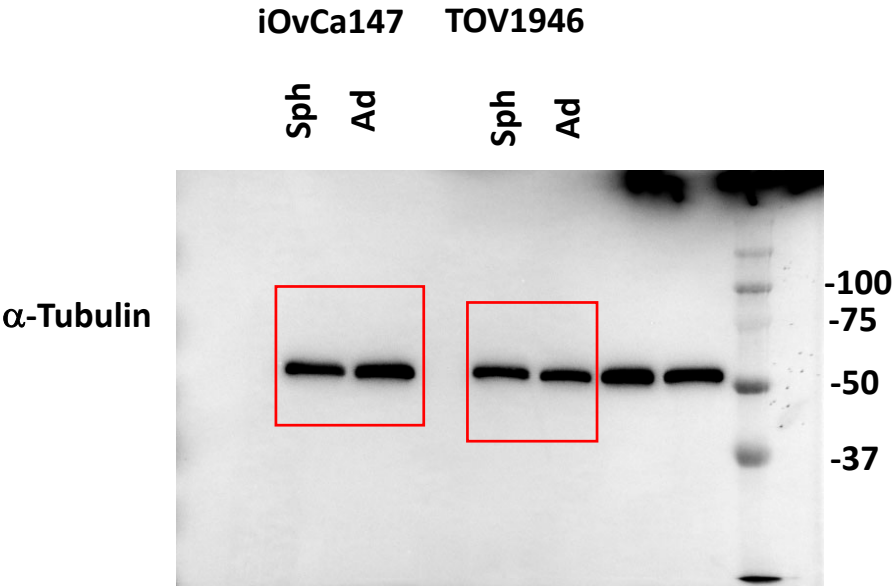

Supplement: Figure 1—source data 2. [file elife-91766-fig1-data2.pdf]

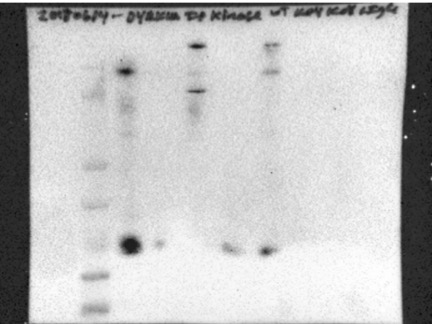

Supplement: Figure 2—figure supplement 1—source data 1. [file elife-91766-fig2-figsupp1-data1.zip › Figure 2 - Figure Supplement 1 - Source Data 1/pTAU S404 F2FS1D.jpg]

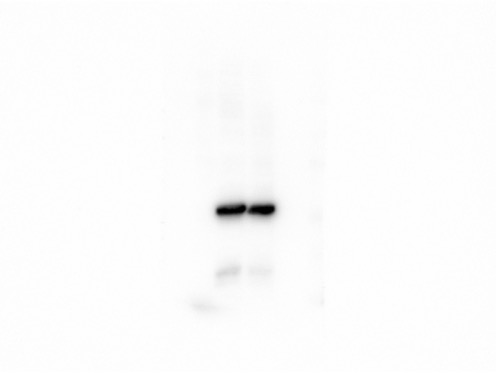

Supplement: Figure 2—figure supplement 1—source data 1. [file elife-91766-fig2-figsupp1-data1.zip › Figure 2 - Figure Supplement 1 - Source Data 1/Tubulin F2FS1C.jpg]

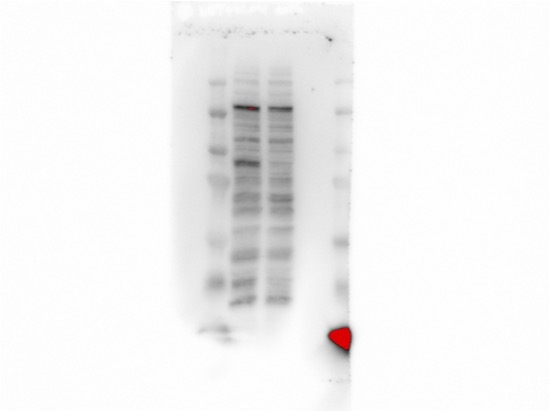

Supplement: Figure 2—figure supplement 1—source data 1. [file elife-91766-fig2-figsupp1-data1.zip › Figure 2 - Figure Supplement 1 - Source Data 1/DYRK1A F2FS1C.jpg]

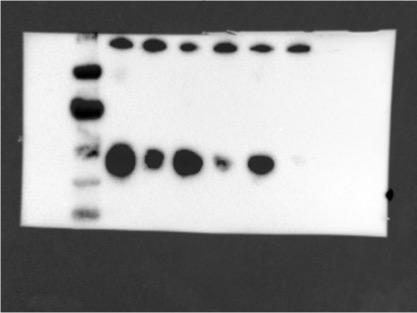

Supplement: Figure 2—figure supplement 1—source data 1. [file elife-91766-fig2-figsupp1-data1.zip › Figure 2 - Figure Supplement 1 - Source Data 1/TAU F2FS1D.jpg]

DYRK1A -

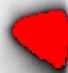

DYRK1A -

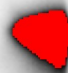

11

11

20180614 - DYADIA Df Kinase WT KOY KOY 15%

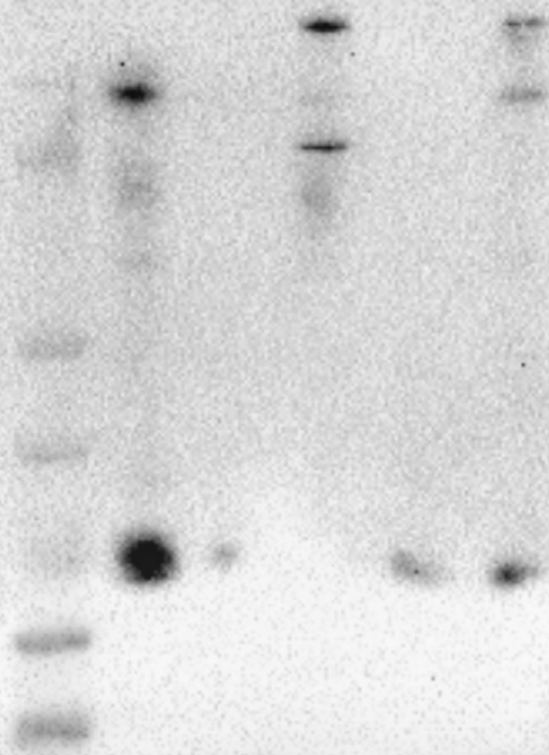

20180614 - DYADIA Df Kinase WT KOY KOY 15%

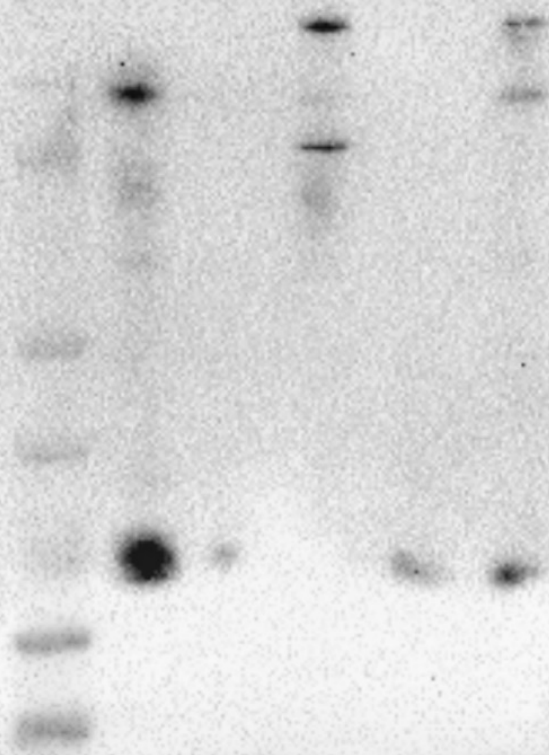

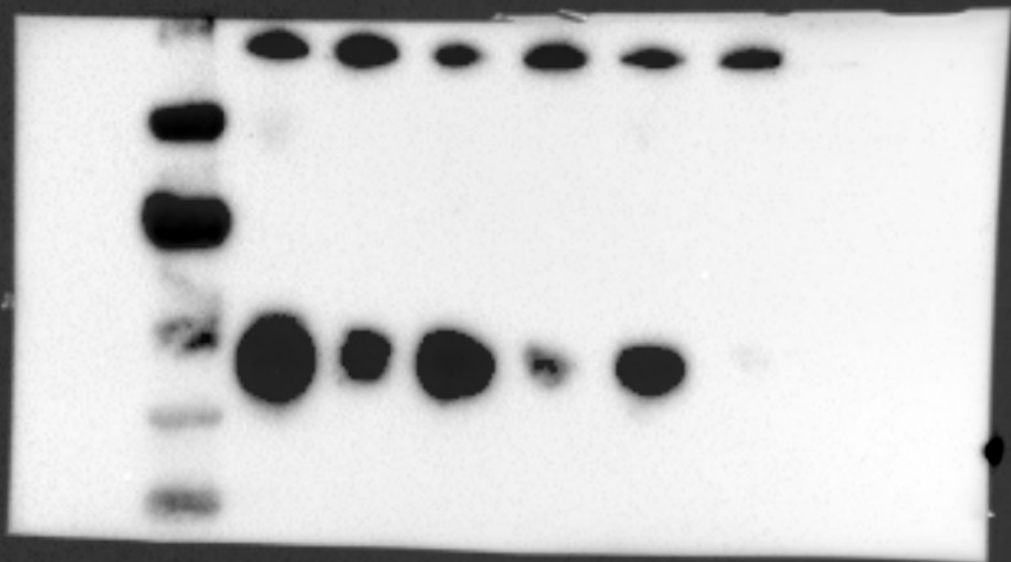

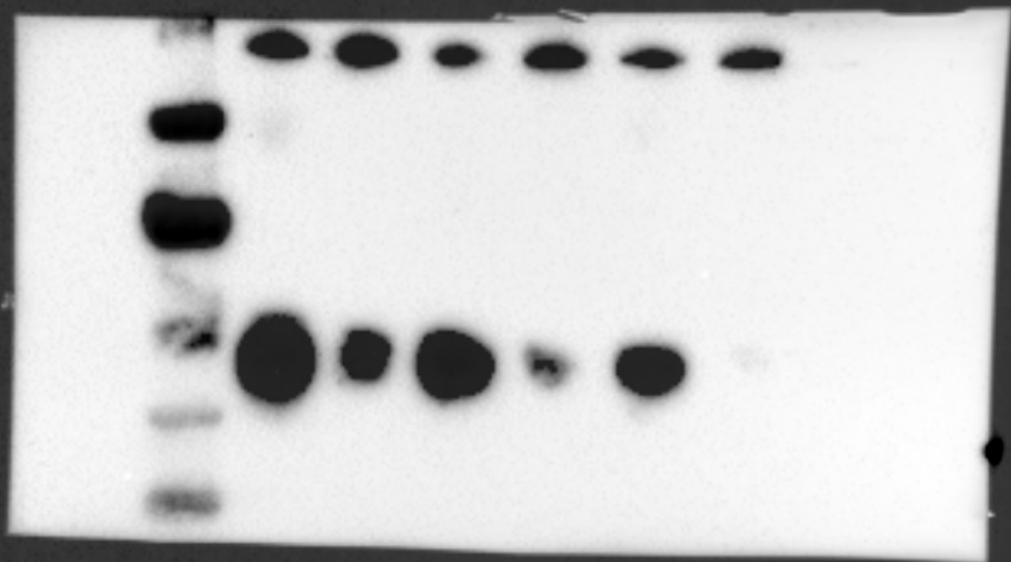

Supplement: Figure 2—figure supplement 1—source data 2. [file elife-91766-fig2-figsupp1-data2.pdf]

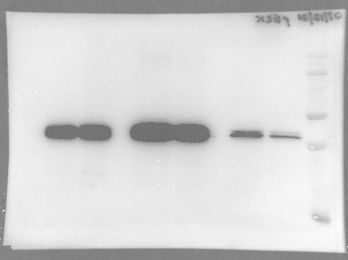

Supplement: Figure 5—source data 2. [file elife-91766-fig5-data2.zip › iOvCa pERK 2021-07-13 14hr 27min_Exposure_12.2sec+user 2021-07-13 14hr 19min.tif]

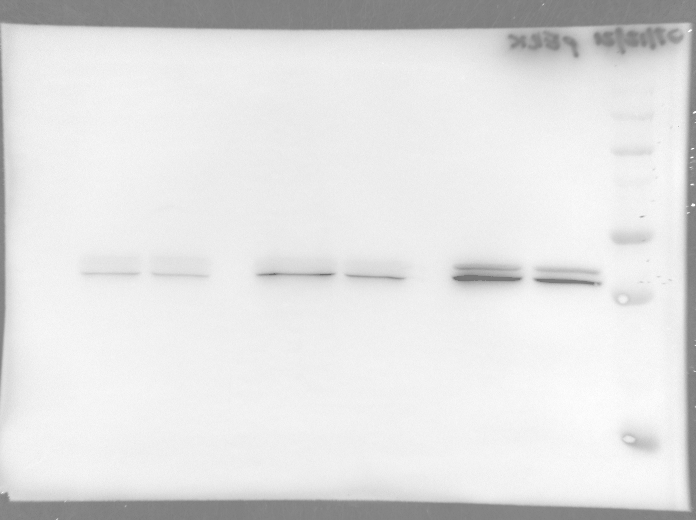

Supplement: Figure 5—source data 2. [file elife-91766-fig5-data2.zip › iOvCa Total ERK 2021-07-14 14hr 46min_Exposure_20.0sec+user 2021-07-14 14hr 40min.tif]

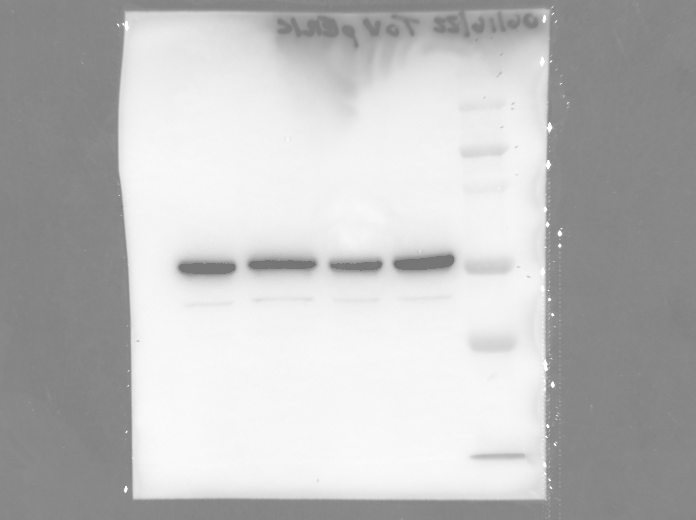

Supplement: Figure 5—source data 2. [file elife-91766-fig5-data2.zip › TOV Tubulin 2022-06-22 14hr 41min_Exposure_3.0sec+user 2022-06-22 14hr 39min.tif]

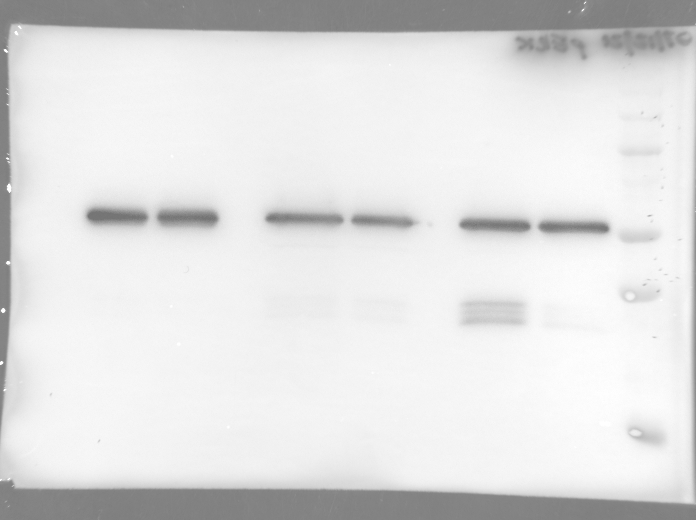

Supplement: Figure 5—source data 2. [file elife-91766-fig5-data2.zip › iOvCa Tubulin 2021-07-23 13hr 48min_Exposure_2.0sec+user 2021-07-23 13hr 47min.tif]

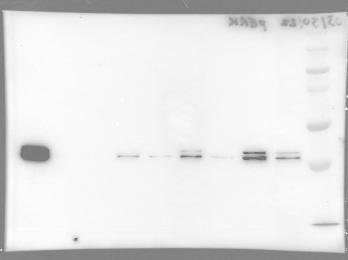

Supplement: Figure 5—source data 2. [file elife-91766-fig5-data2.zip › OV8 pERK 2022-03-31 14hr 18min-1_Exposure_15.0sec+user 2022-03-31 14hr 18min.tif]

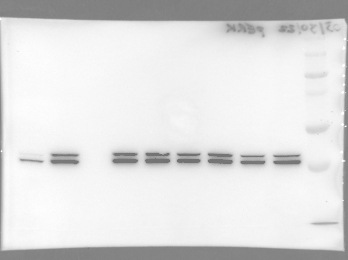

Supplement: Figure 5—source data 2. [file elife-91766-fig5-data2.zip › OV8 Total ERK 2022-04-01 12hr 34min_Exposure_5.0sec+user 2022-04-01 12hr 33min.tif]

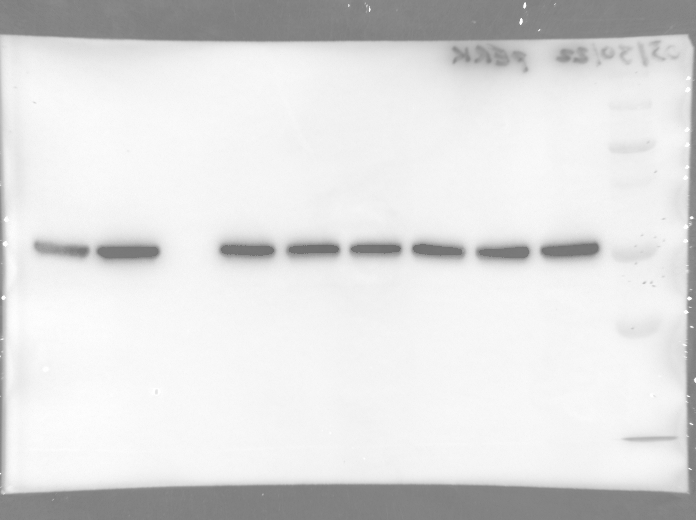

Supplement: Figure 5—source data 2. [file elife-91766-fig5-data2.zip › OV8 Tubulin 2022-05-05 15hr 19min-1_Exposure_10.0sec+user 2022-05-05 15hr 19min.tif]

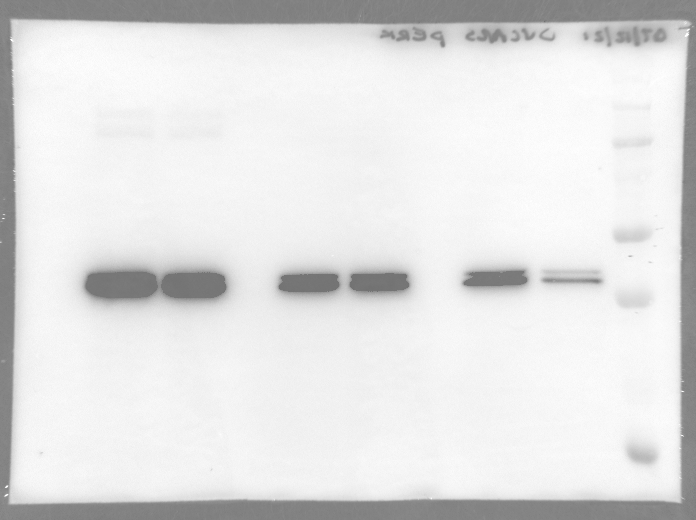

Supplement: Figure 5—source data 2. [file elife-91766-fig5-data2.zip › OVCAR3 pERK 2021-07-13 14hr 08min_Exposure_17.0sec+user 2021-07-13 14hr 05min.tif]

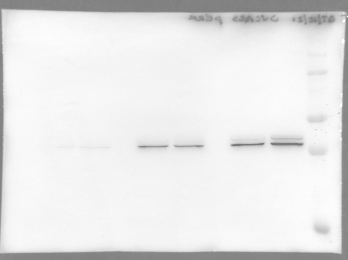

Supplement: Figure 5—source data 2. [file elife-91766-fig5-data2.zip › OVCAR3 Total ERK 2021-07-14 14hr 21min_Exposure_2.0sec+user 2021-07-14 14hr 20min.tif]

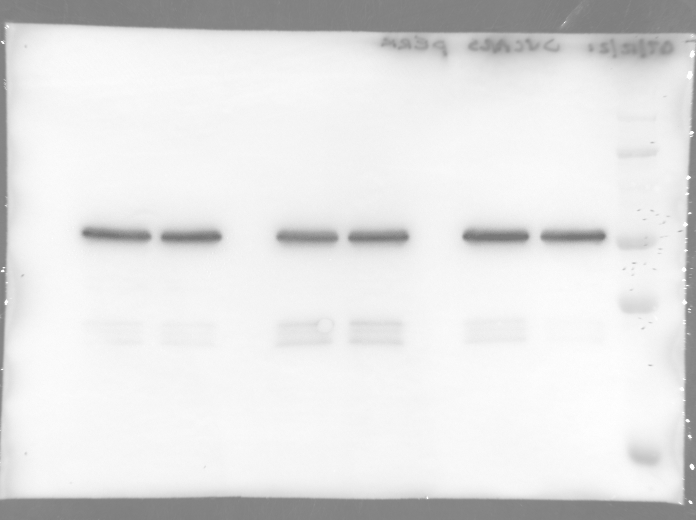

Supplement: Figure 5—source data 2. [file elife-91766-fig5-data2.zip › OVCAR3 tubulin 2021-07-23 13hr 39min-1_Exposure_2.0sec+user 2021-07-23 13hr 39min.tif]

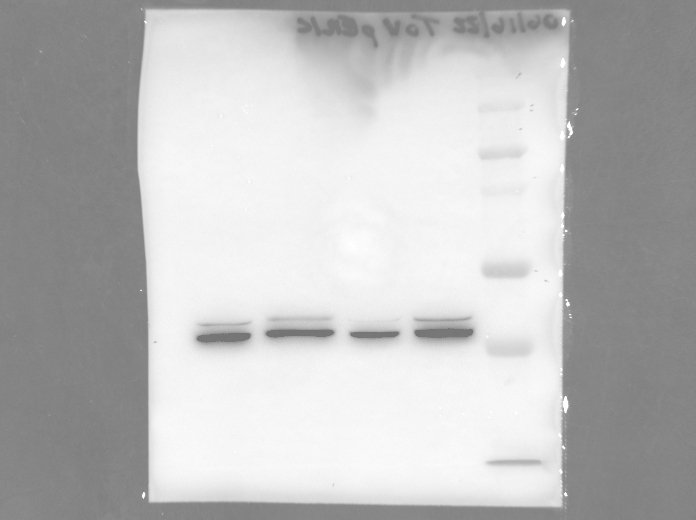

Supplement: Figure 5—source data 2. [file elife-91766-fig5-data2.zip › Total ERK TOV 2022-06-21 13hr 25min_Exposure_15.0sec+Template.tif]

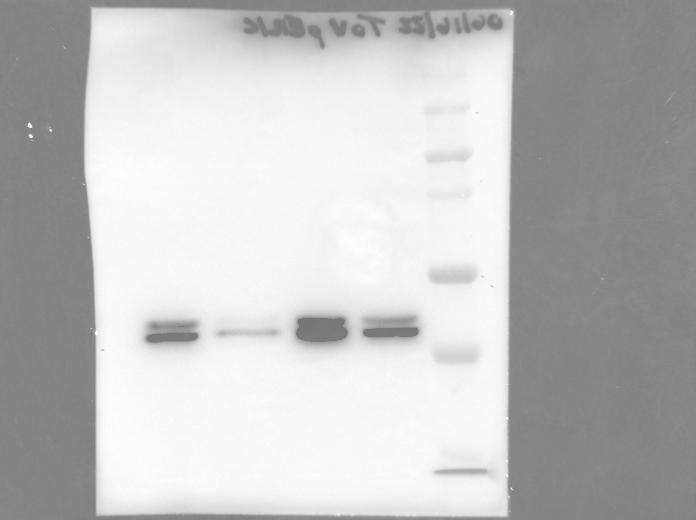

Supplement: Figure 5—source data 2. [file elife-91766-fig5-data2.zip › TOV pERK HR NTN1 2022-06-17 15hr 23min_Exposure_16.0sec+Template.tif]

Fig. 5C

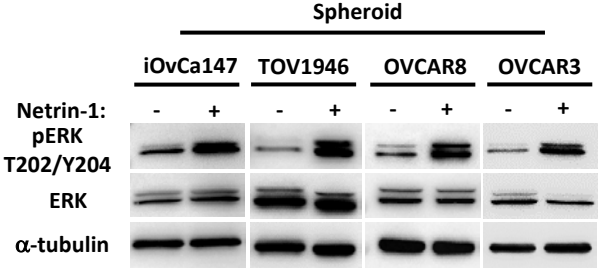

Fig. 5C

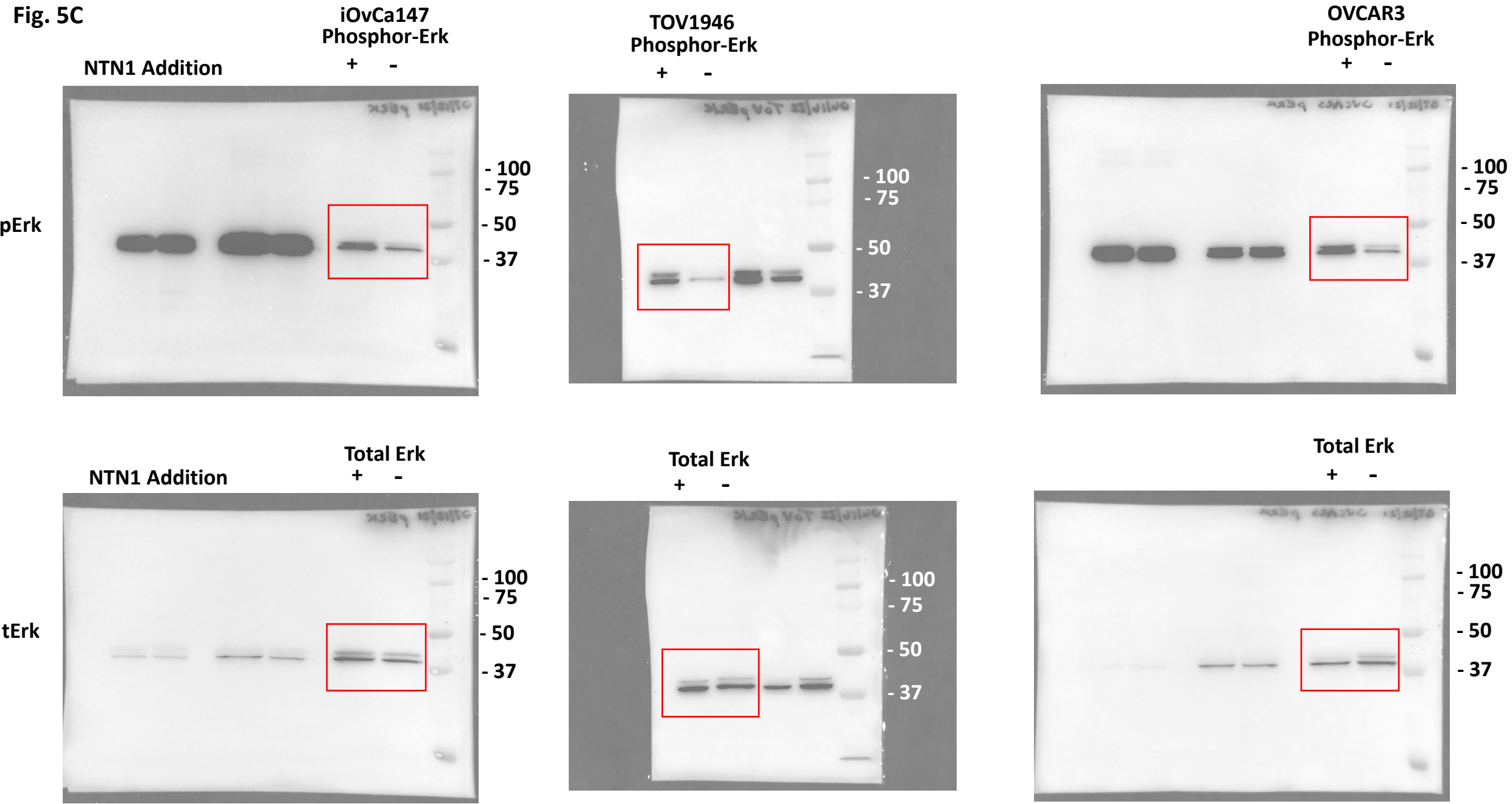

Fig. 5C

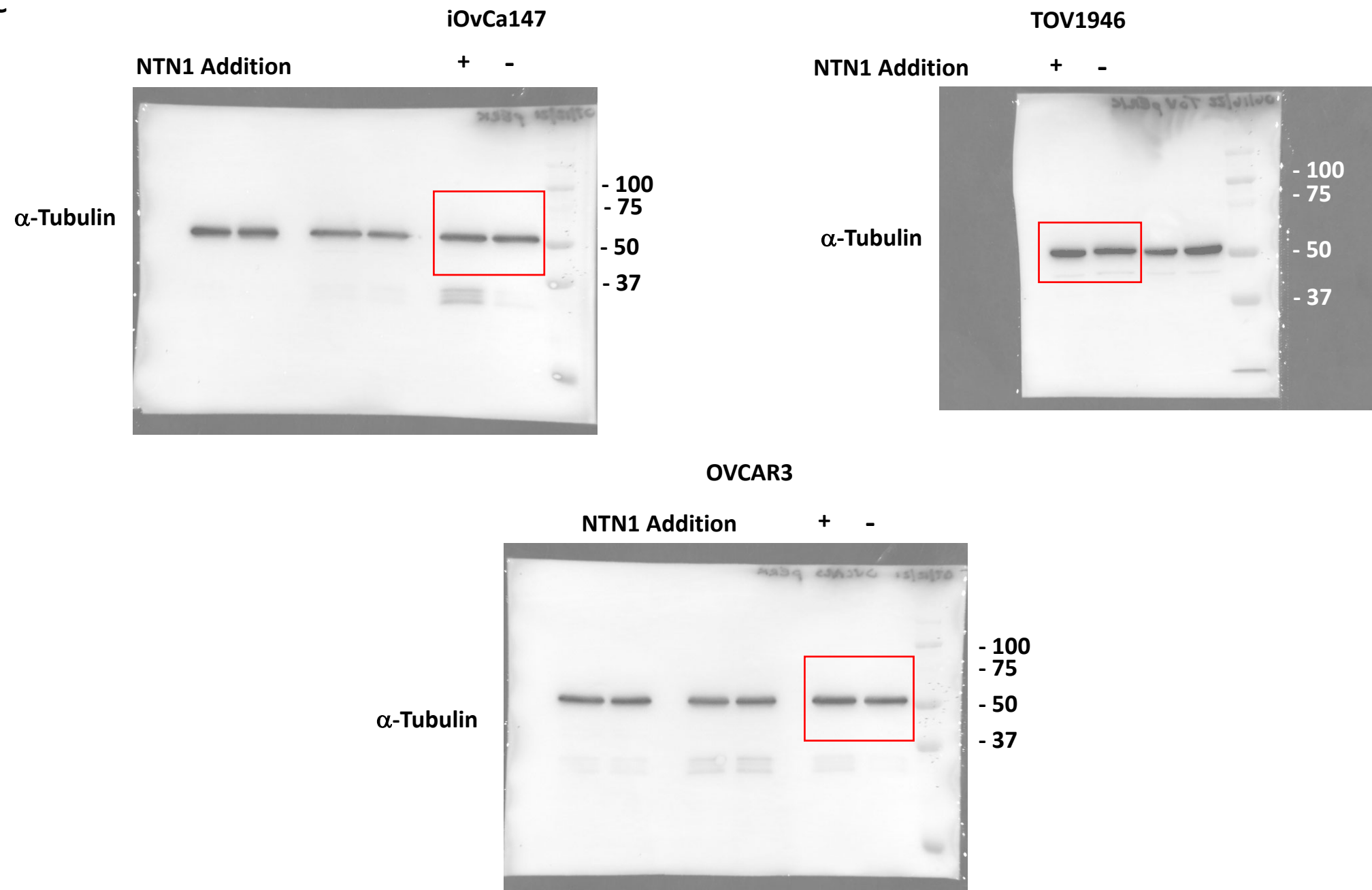

Fig. 5C

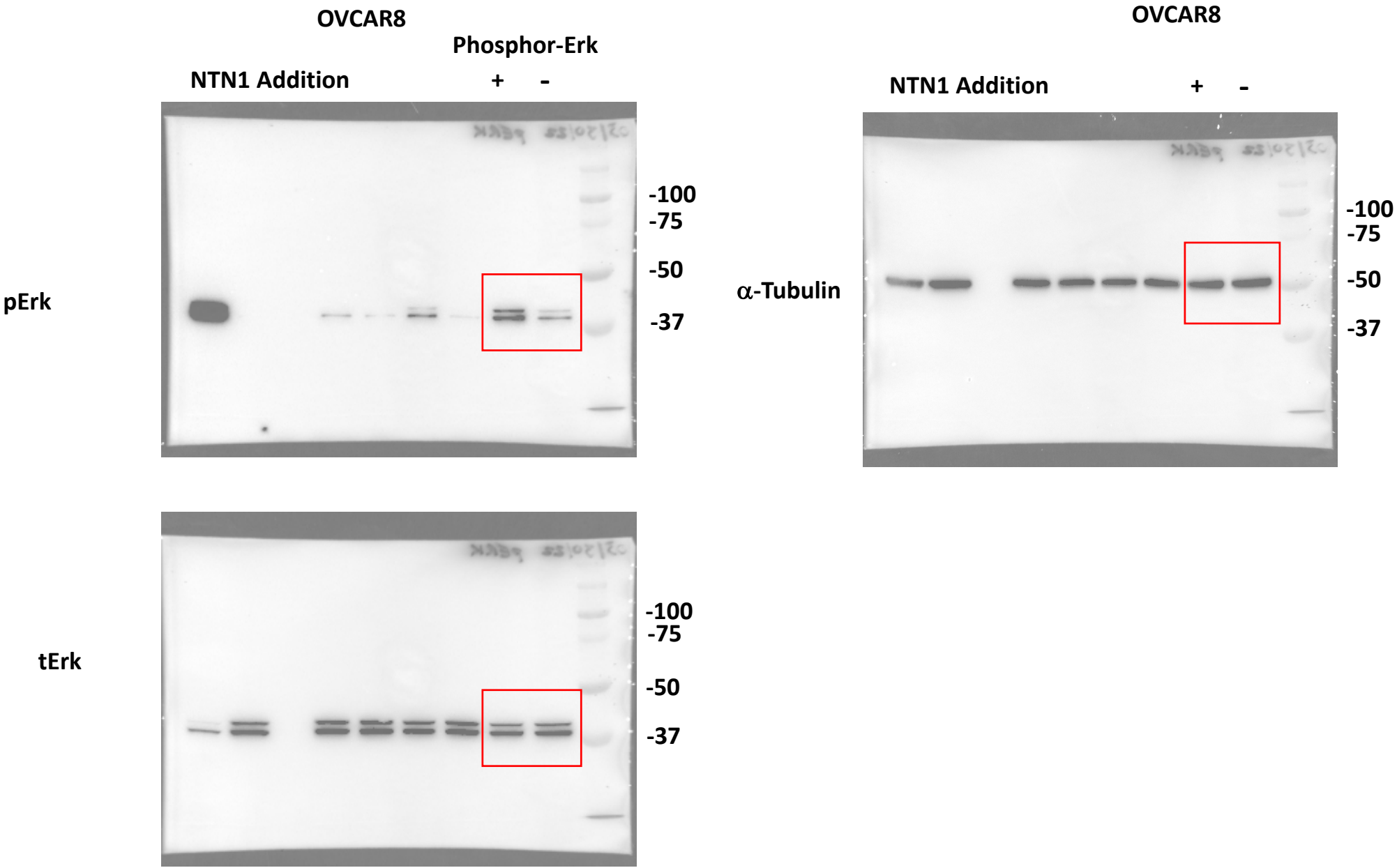

Supplement: Figure 5—source data 3. [file elife-91766-fig5-data3.pdf]

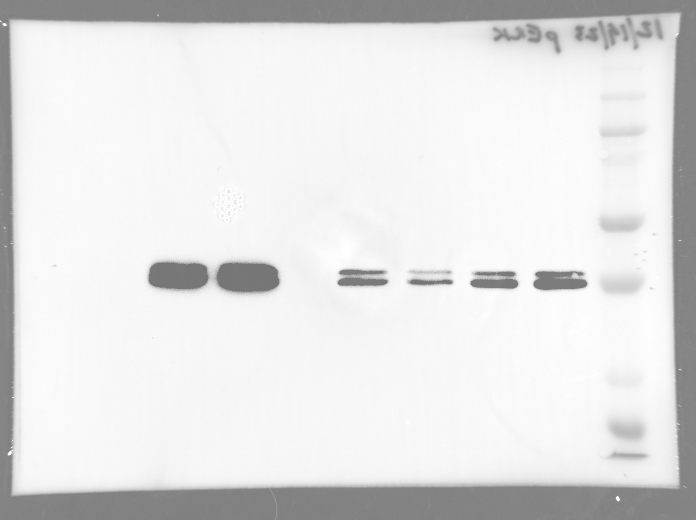

Supplement: Figure 5—source data 4. [file elife-91766-fig5-data4.zip › pErk 2023-12-20 14hr 04min_Exposure_12.0sec+Template for pErk.tif]

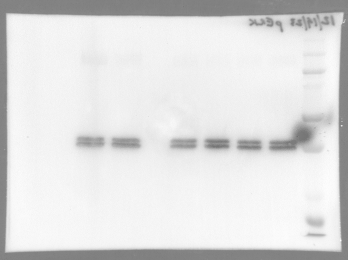

Supplement: Figure 5—source data 4. [file elife-91766-fig5-data4.zip › Total Erk 2023-12-21 15hr 14min_Exposure_20.0sec+user 2023-12-21 15hr 10min.tif]

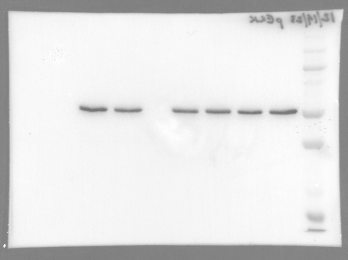

Supplement: Figure 5—source data 4. [file elife-91766-fig5-data4.zip › Tubulin 2023-12-22 13hr 03min_Exposure_6.0sec+user 2023-12-22 13hr 02min.tif]

Fig. 5D

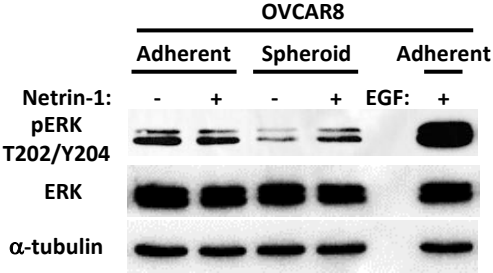

Fig. 5D

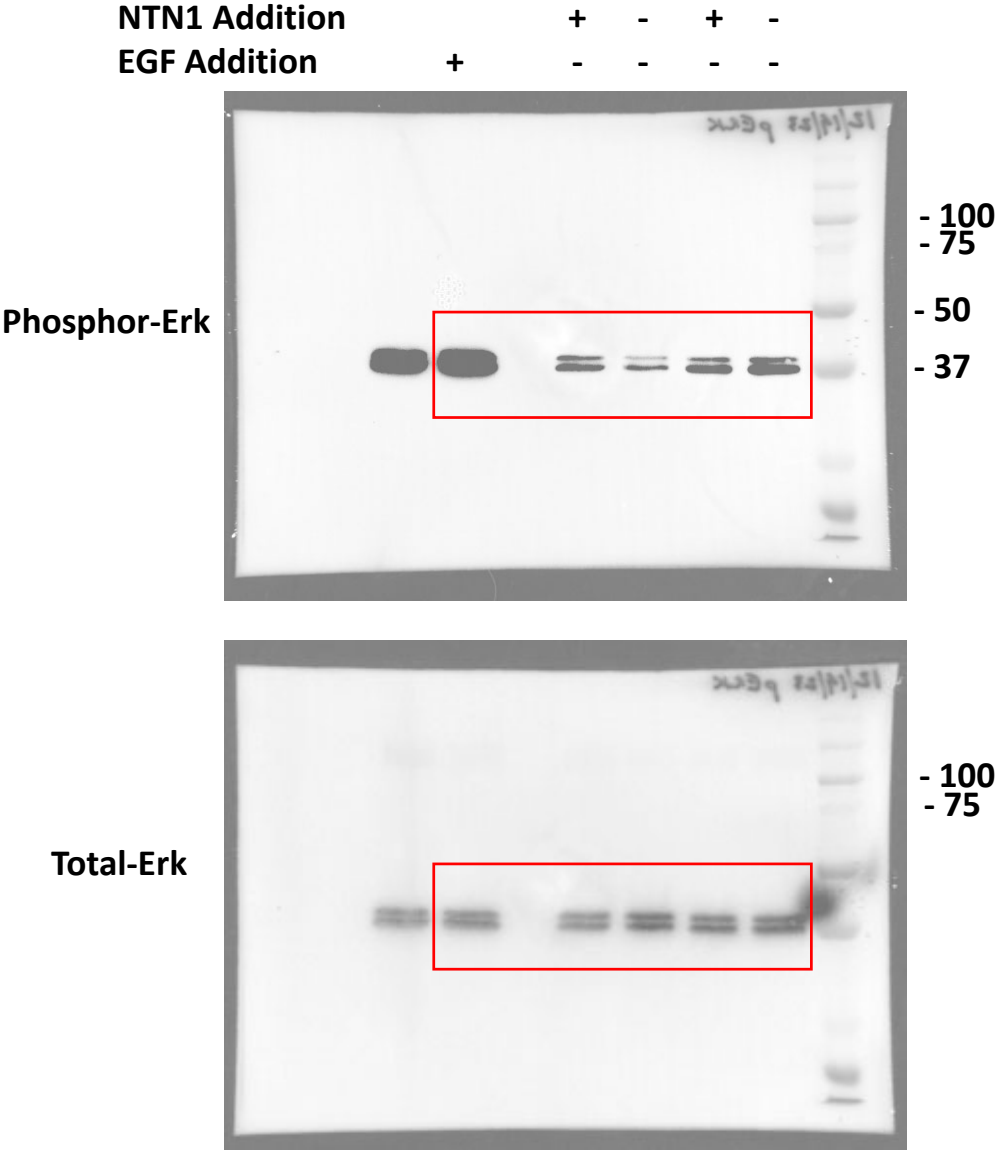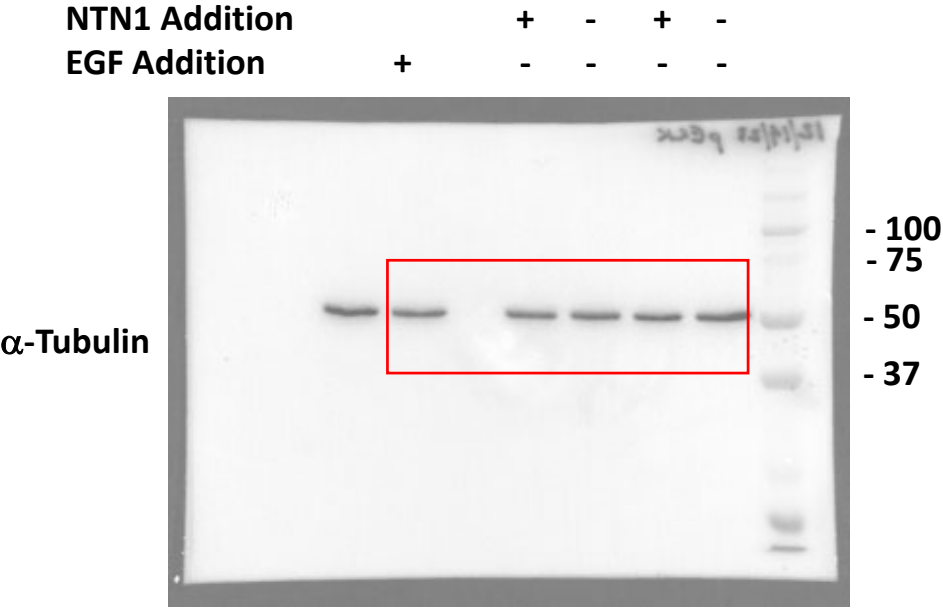

Supplement: Figure 5—source data 5. [file elife-91766-fig5-data5.pdf]

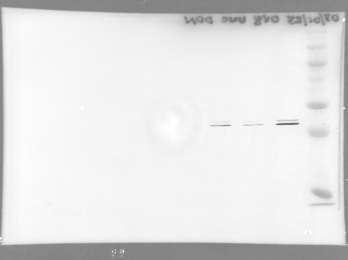

Supplement: Figure 5—source data 6. [file elife-91766-fig5-data6.zip › OV8 pERK 2023-03-02 13hr 33min_Exposure_4.0sec+template for pErk.tif]

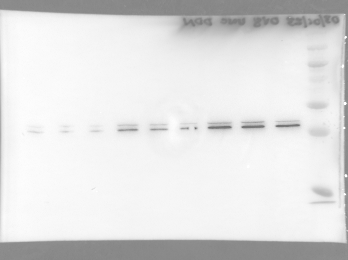

Supplement: Figure 5—source data 6. [file elife-91766-fig5-data6.zip › Total erk2023-03-03 14hr 46min-1_Exposure_2.0sec+Template for Total Erk.tif]

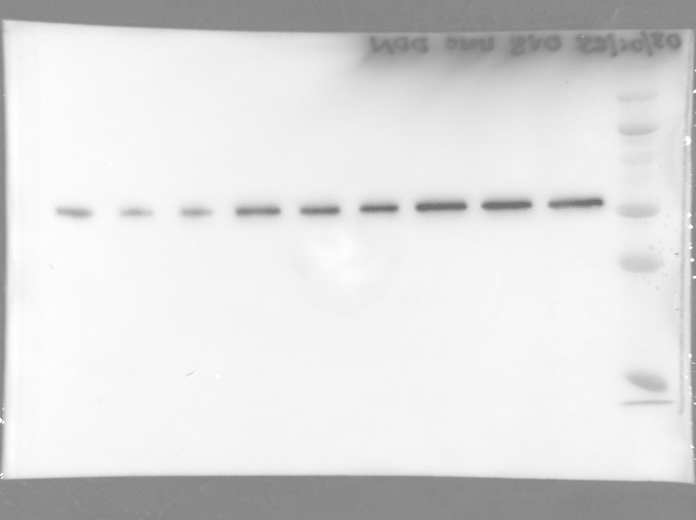

Supplement: Figure 5—source data 6. [file elife-91766-fig5-data6.zip › Tubulin 2023-03-06 15hr 28min_Exposure_4.0sec+template for tubulin.tif]

**Fig. 5E**

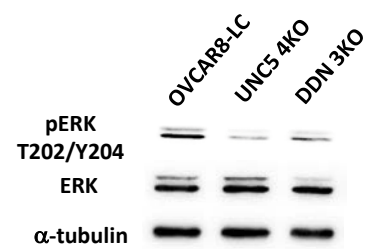

Fig. 5E

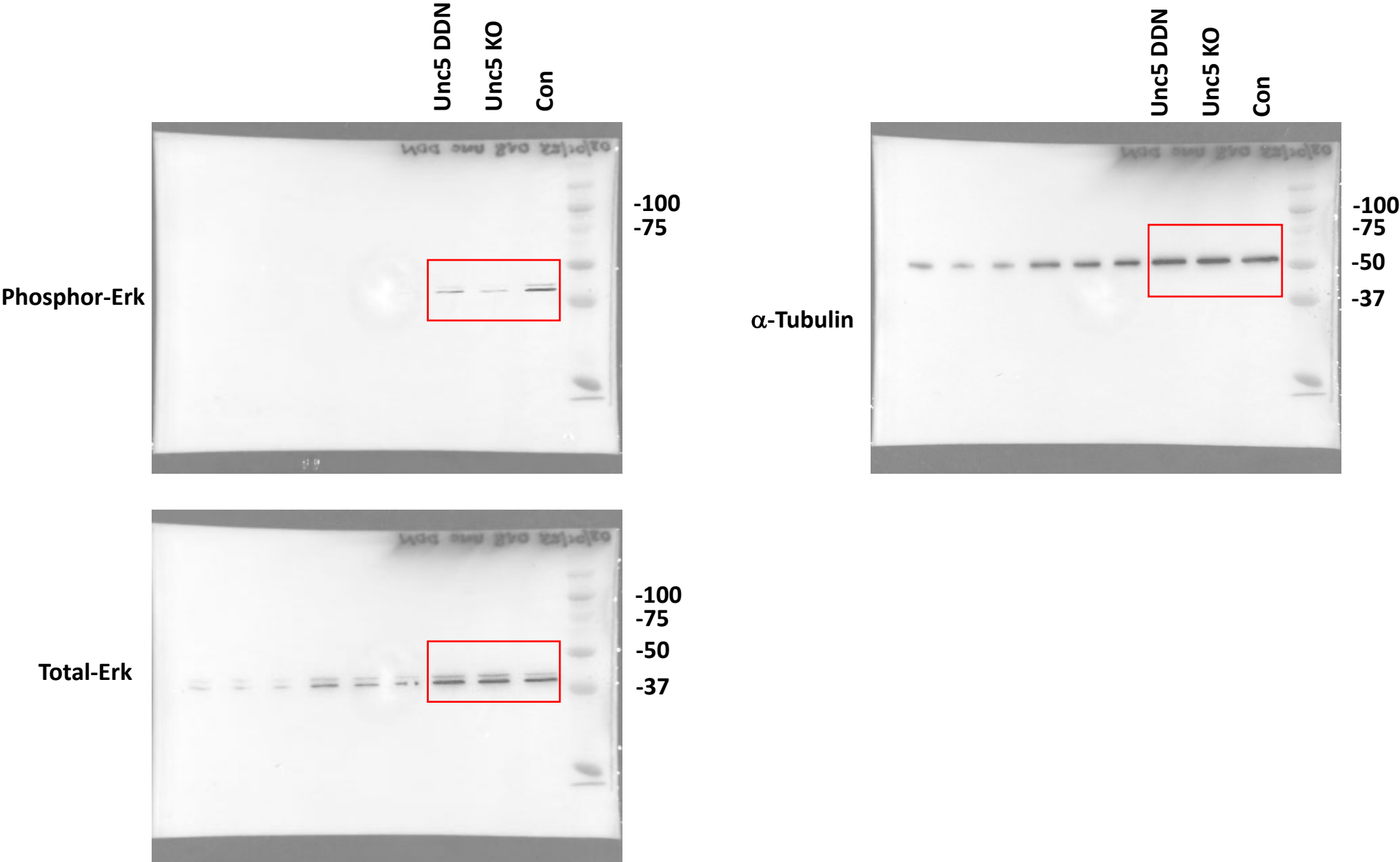

Supplement: Figure 5—source data 7. [file elife-91766-fig5-data7.pdf]

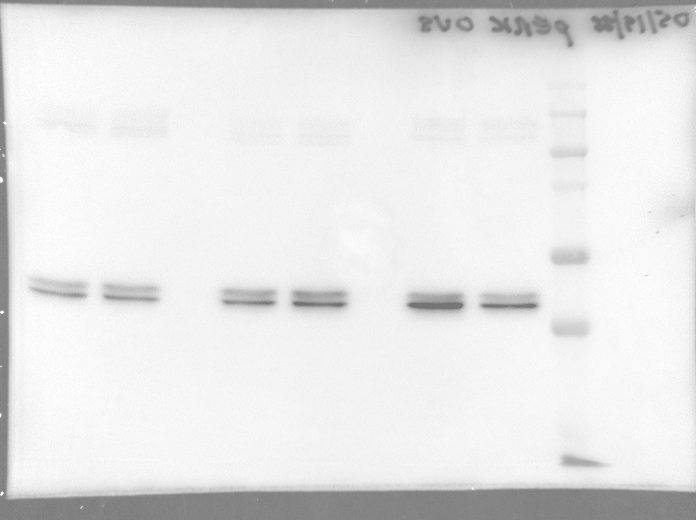

Supplement: Figure 5—source data 8. [file elife-91766-fig5-data8.zip › OV8 UNC5 KO_DDN NTN12022-05-20 15hr 55min_Exposure_18.0sec+user 2022-05-20_E.tif]

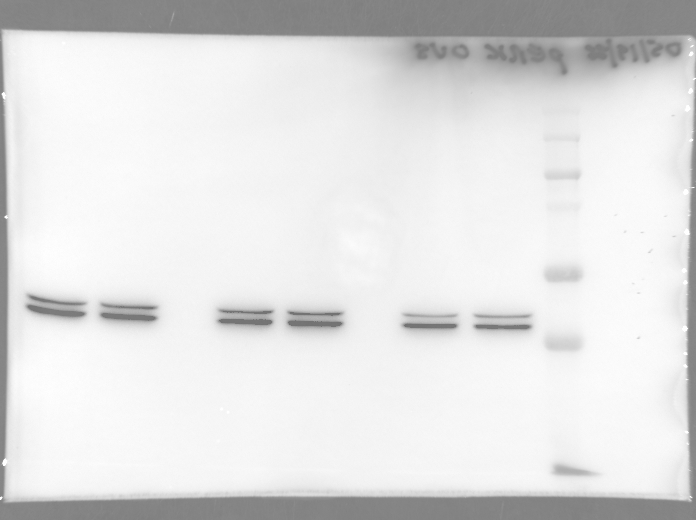

Supplement: Figure 5—source data 8. [file elife-91766-fig5-data8.zip › Total ERK Con_UNC5 KO_DDN 2022-05-25 14hr 06min_Exposure_8.0sec+user 2022-05-25 14hr 05min.tif]

Fig. 5F

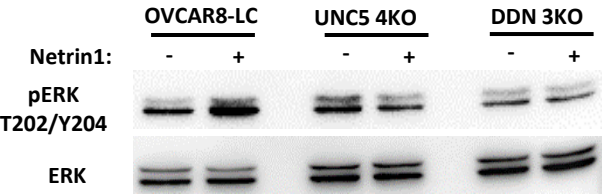

Fig. 5F

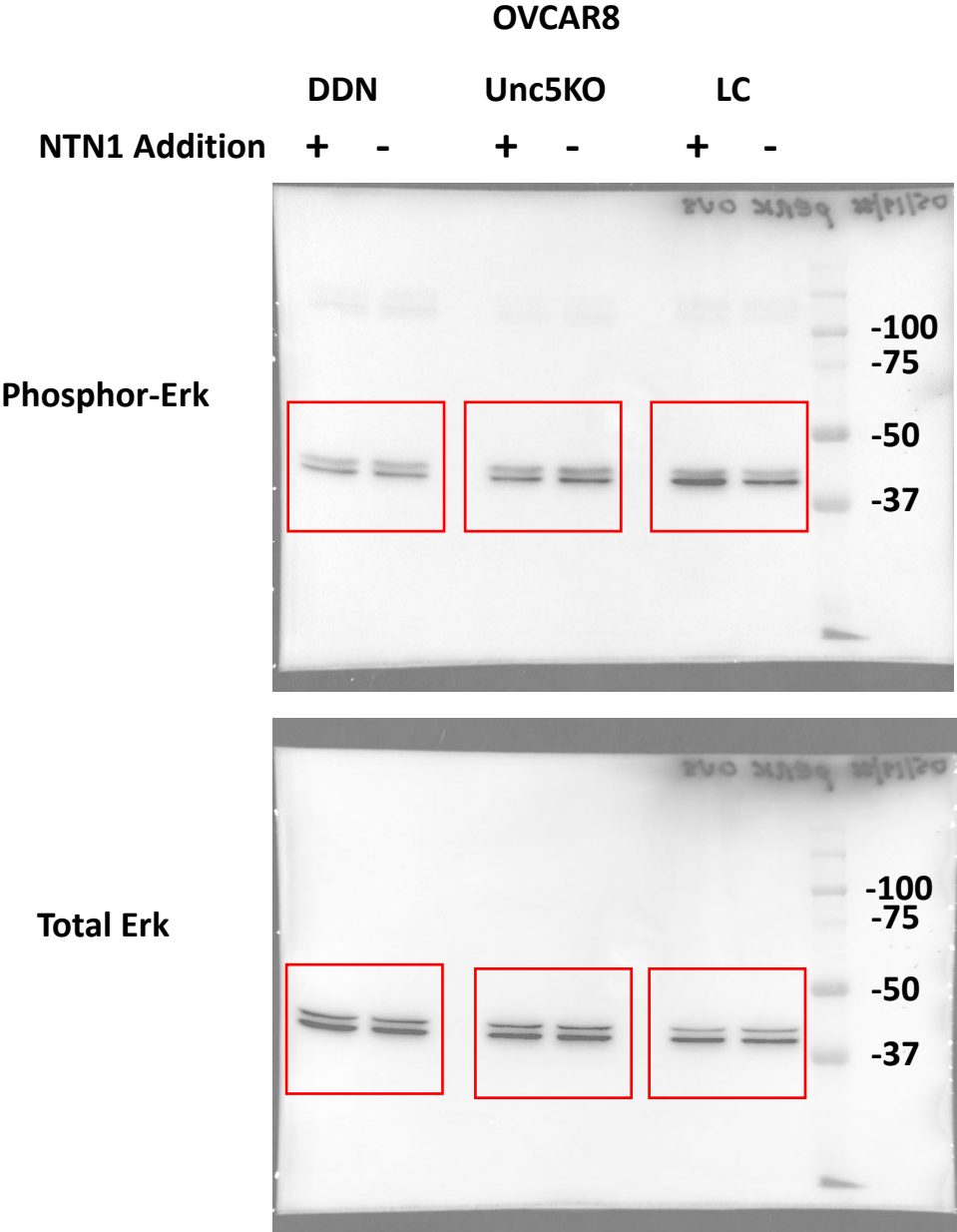

Supplement: Figure 5—source data 9. [file elife-91766-fig5-data9.pdf]

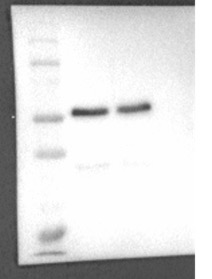

Supplement: Figure 5—source data 11. [file elife-91766-fig5-data11.zip › Figure5-Source Data11/Tubulin-F5I.jpg]

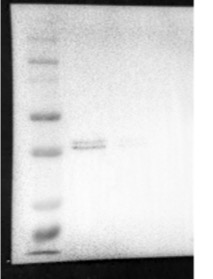

Supplement: Figure 5—source data 11. [file elife-91766-fig5-data11.zip › Figure5-Source Data11/pERK-F5I.jpg]

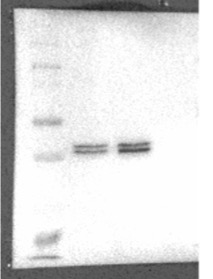

Supplement: Figure 5—source data 11. [file elife-91766-fig5-data11.zip › Figure5-Source Data11/ERK-F5I.jpg]

**Fig. 5I**

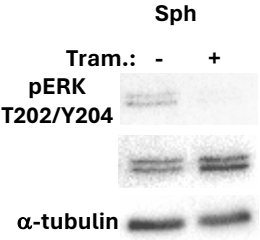

Fig. 5I

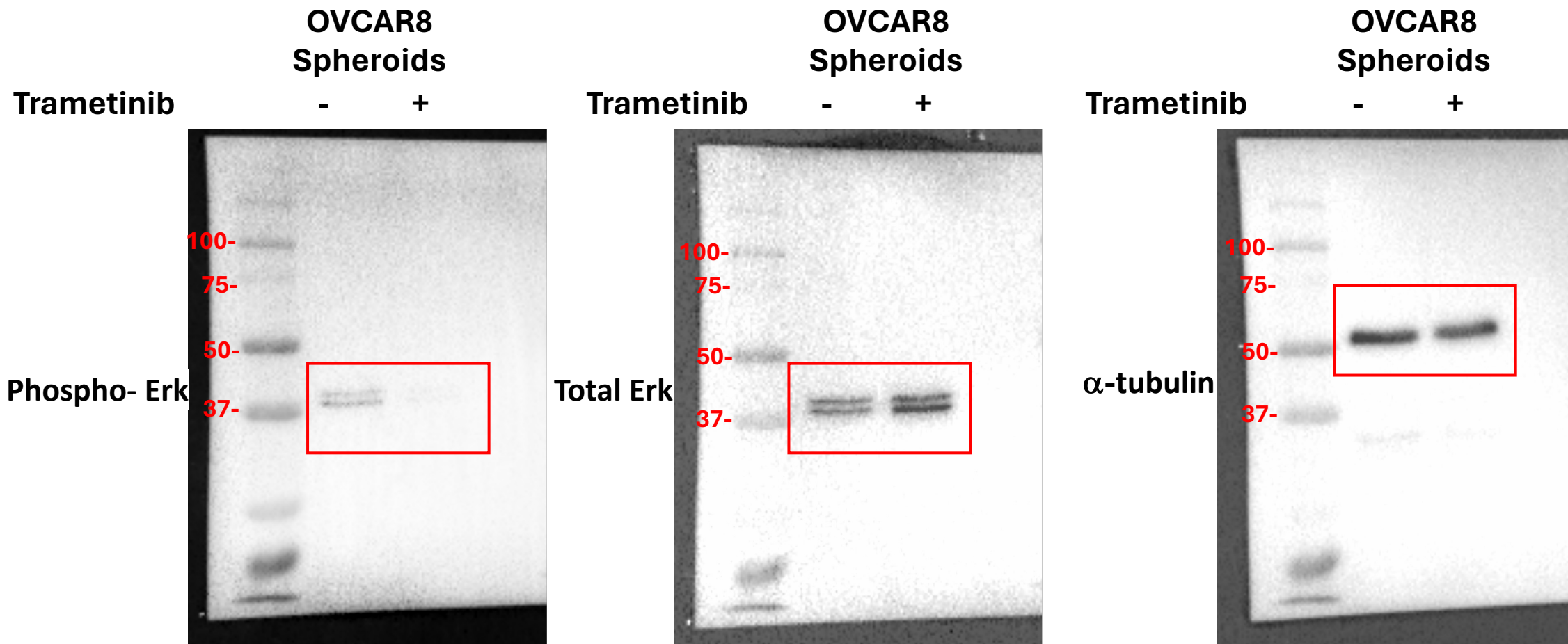

Supplement: Figure 5—source data 12. [file elife-91766-fig5-data12.pdf]

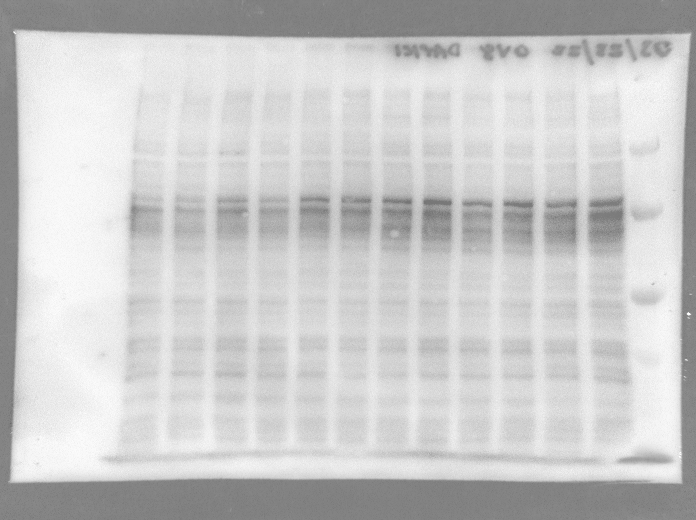

Supplement: Figure 5—figure supplement 1—source data 2. [file elife-91766-fig5-figsupp1-data2.zip › OV8 pDAPK1 2022-03-24 14hr 17min_Exposure_2.0sec+user 2022-03-24 14hr 16min.tif]

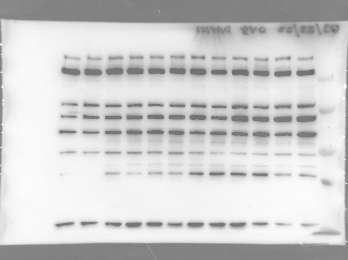

Supplement: Figure 5—figure supplement 1—source data 2. [file elife-91766-fig5-figsupp1-data2.zip › OV8 T_DAPK1 2022-03-25 14hr 36min_Exposure_8.0sec+user 2022-03-25 14hr 35min.tif]

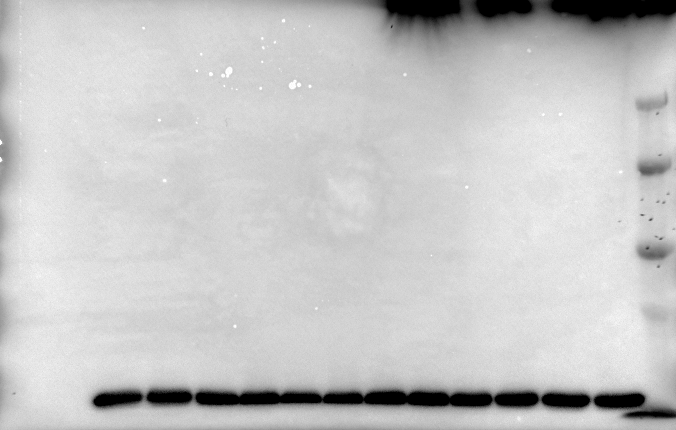

Supplement: Figure 5—figure supplement 1—source data 2. [file elife-91766-fig5-figsupp1-data2.zip › OV8_Tubulin 2022-03-29 14hr 10min-1_Exposure_2.0sec+user 2022-03-29 14hr 10min.tif]

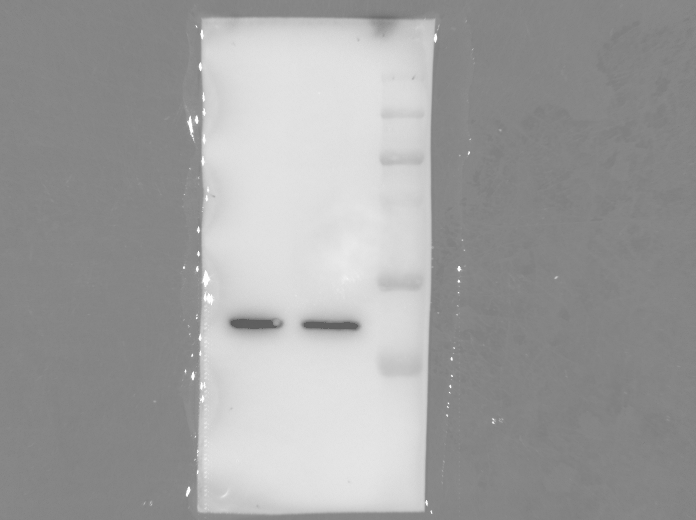

Supplement: Figure 7—source data 1. [file elife-91766-fig7-data1.zip › Actin 2022-11-11 15hr 18min_Exposure_10.0sec+user 2022-11-11 15hr 17min.tif]

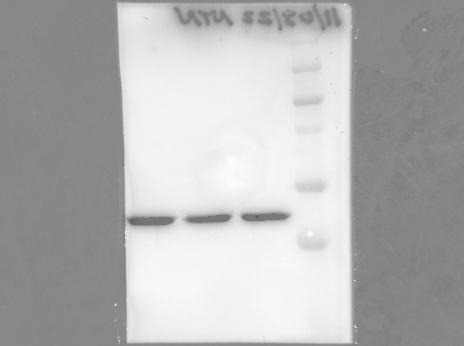

Supplement: Figure 7—source data 1. [file elife-91766-fig7-data1.zip › Actin Myc 2022-11-10 15hr 06min_Exposure_1.0sec+user 2022-11-10 15hr 03min.tif]

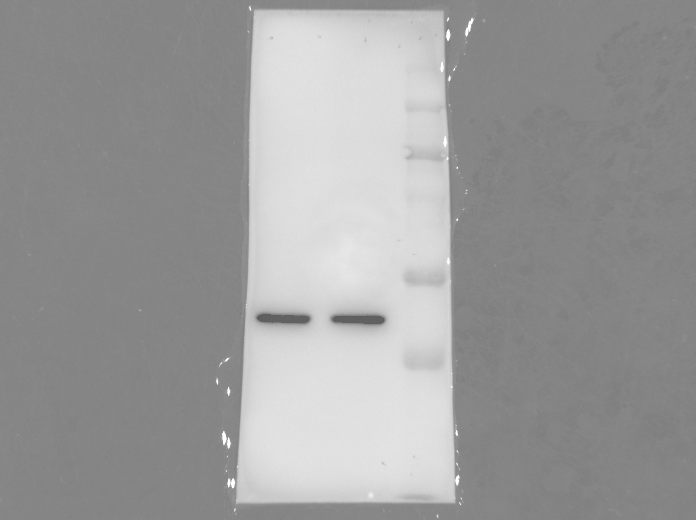

Supplement: Figure 7—source data 1. [file elife-91766-fig7-data1.zip › Actin_B 2022-11-11 15hr 24min_Exposure_11.0sec+user 2022-11-11 15hr 22min.tif]

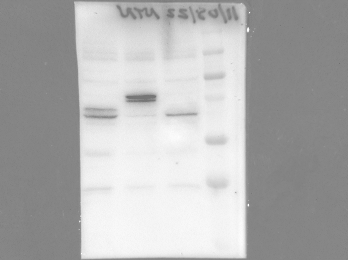

Supplement: Figure 7—source data 1. [file elife-91766-fig7-data1.zip › Myc Ab 2022-11-09 15hr 27min_Exposure_20.0sec+Template.tif]

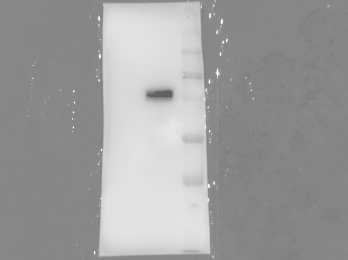

Supplement: Figure 7—source data 1. [file elife-91766-fig7-data1.zip › NTN1 Ab 2022-11-10 14hr 58min_Exposure_2.0sec+user 2022-11-10 14hr 57min.tif]

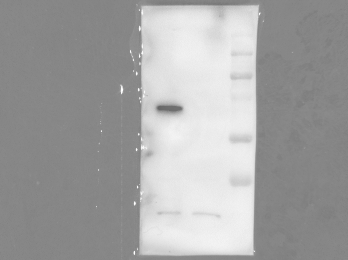

Supplement: Figure 7—source data 1. [file elife-91766-fig7-data1.zip › NTN3 Ab 2022-11-10 14hr 52min-1_Exposure_5.0sec+Template for NTN3 Ab.tif]

Fig.7B

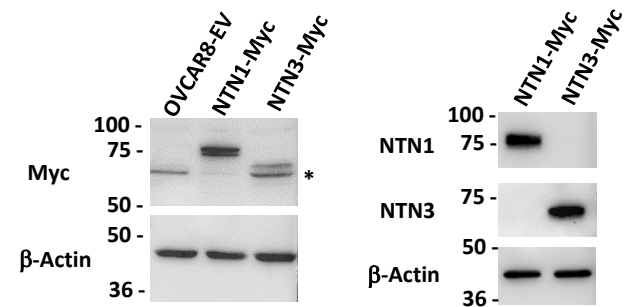

Fig.7B

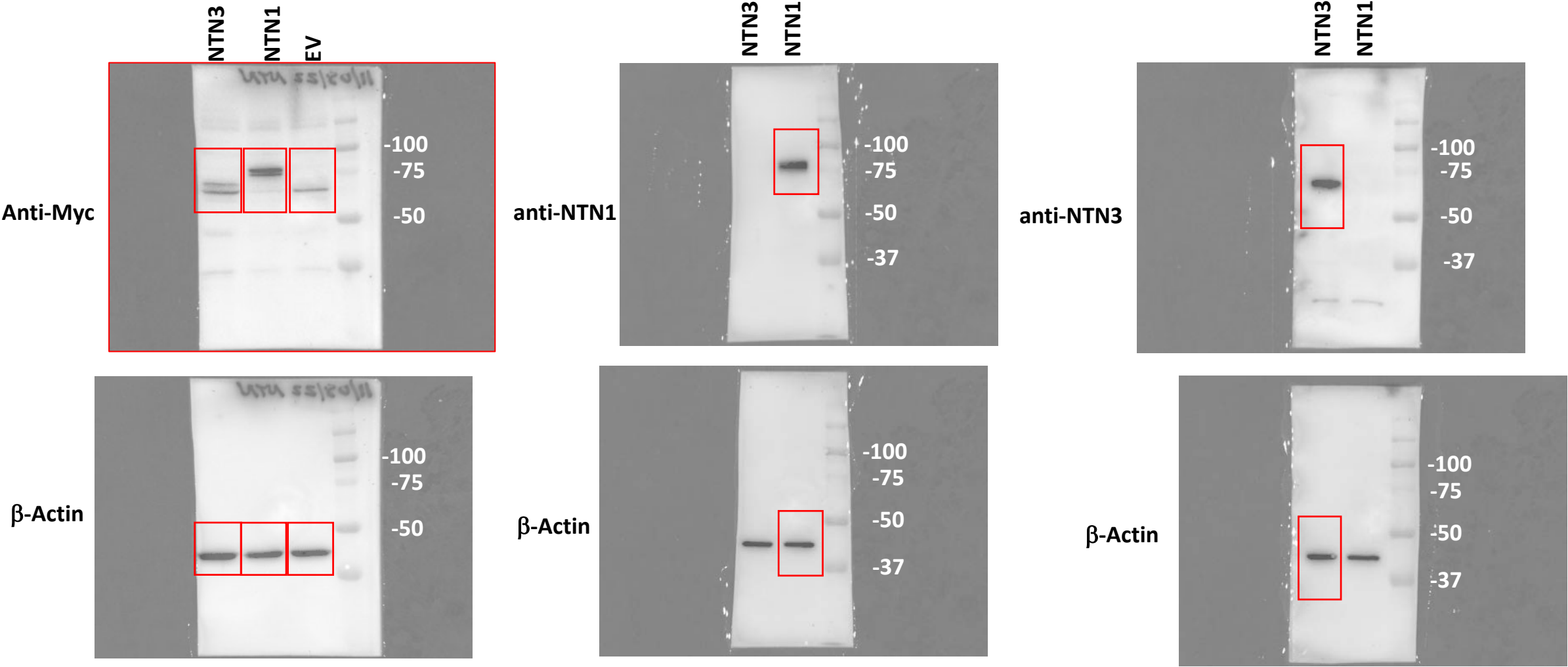

Supplement: Figure 7—source data 2. [file elife-91766-fig7-data2.pdf]
