# Supplementary material for: Netrin signaling mediates survival of dormant epithelial ovarian cancer cells
Source: eLife. 2024 Jul 18;12:RP91766. doi: 10.7554/eLife.91766 (PMC11257678; doi:10.7554/eLife.91766)

Figure 5D – Figure Supplement 1

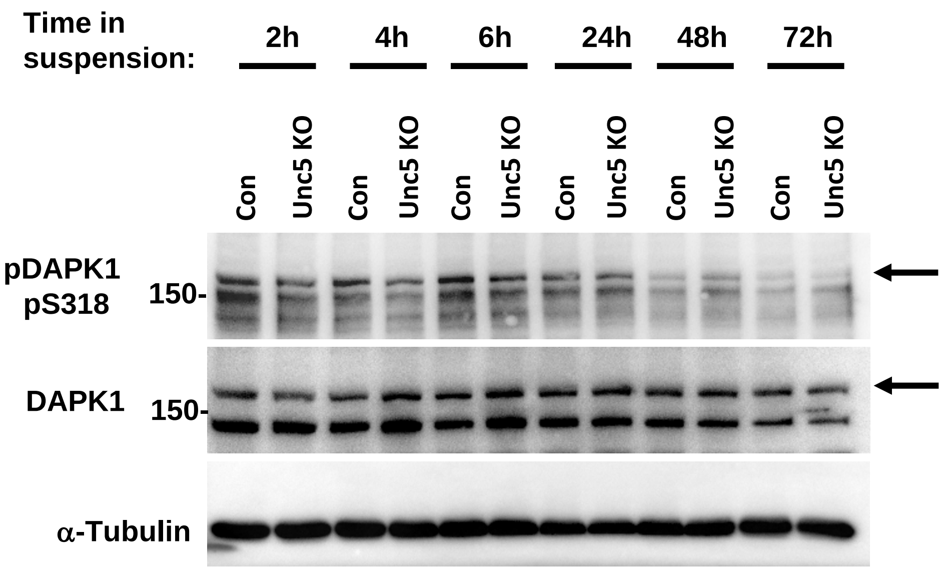

Figure [illegible]D – Figure Supplement 1

Fig.1A Source Data 2.pdf

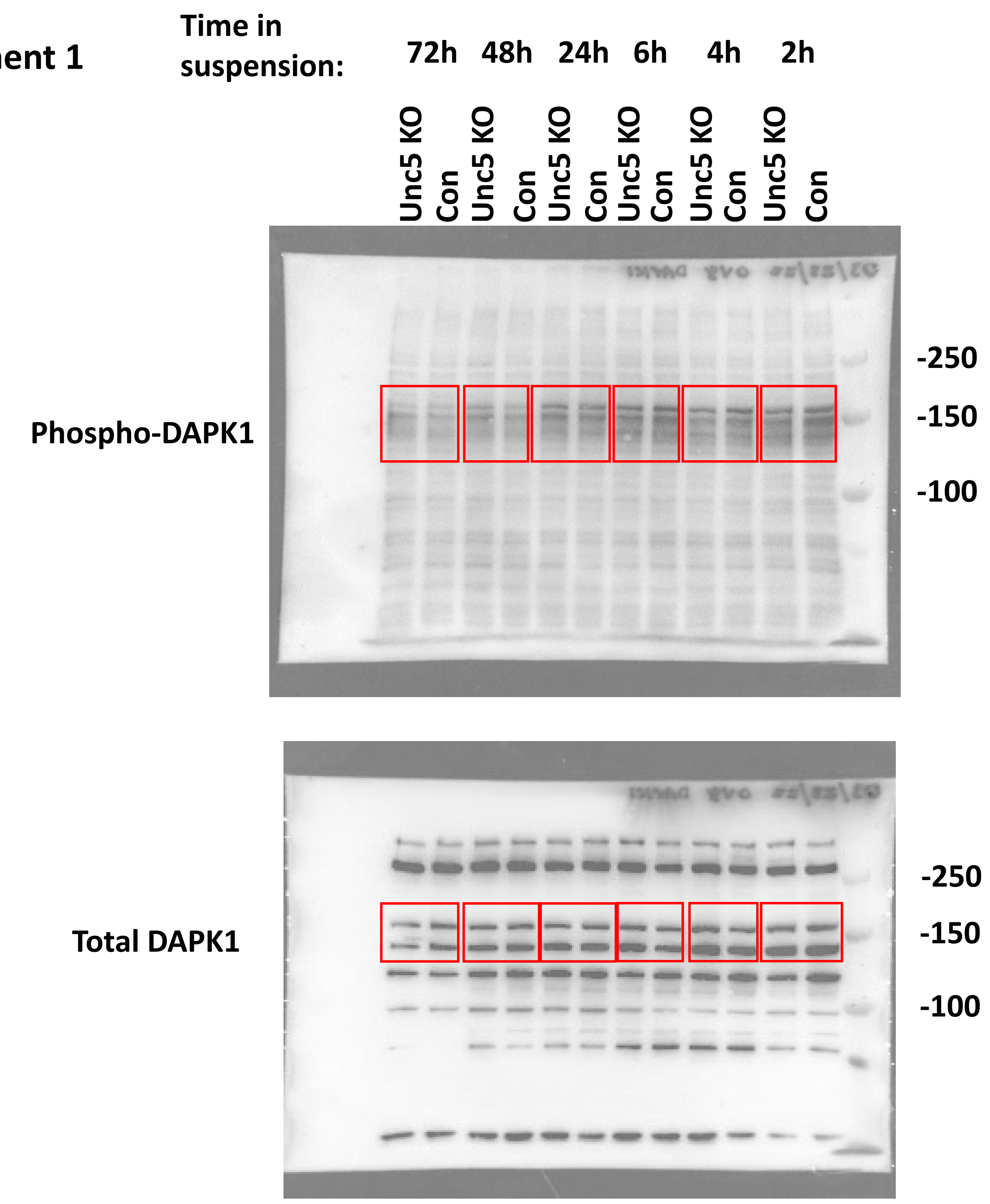

Figure 5D – Figure Supplement 1

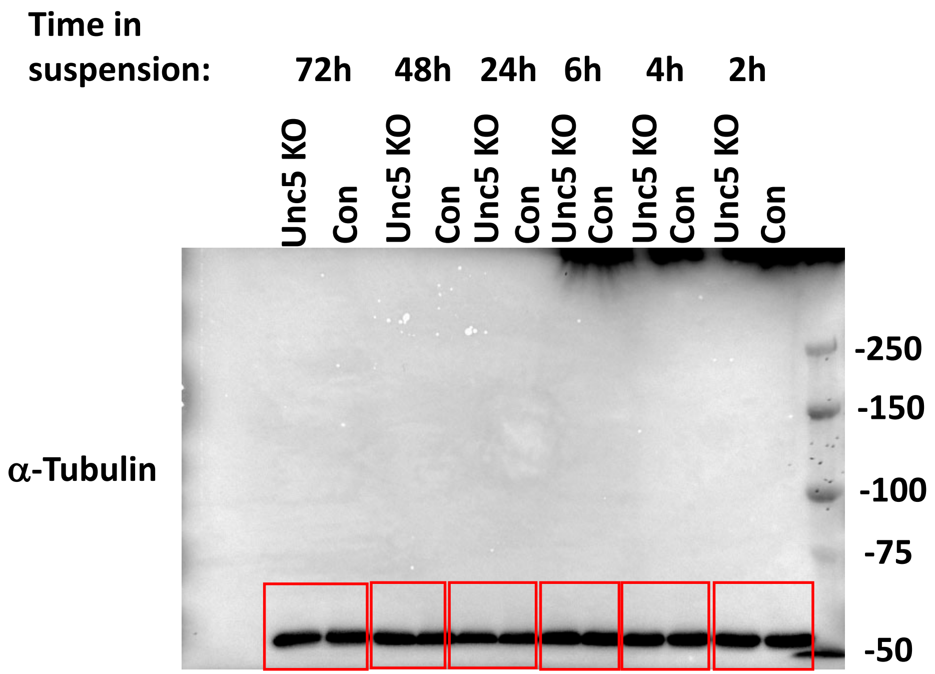

Supplement: Figure 5—figure supplement 1—source data 3. [file elife-91766-fig5-figsupp1-data3.pdf]
